# Insights into the role of crustose coralline algae microbiomes on coral larval settlement in the Great Barrier Reef

**DOI:** 10.1186/s40793-026-00907-6

**Published:** 2026-05-09

**Authors:** Abigail C. Turnlund, Paul A. O’Brien, Laura Rix, Sophie Ferguson, Nicole S. Webster, Guillermo Diaz-Pulido, Muhammad Abdul Wahab, Miguel Lurgi, Inka Vanwonterghem

**Affiliations:** 1 Australian Centre for Ecogenomics, The University of Queensland, Brisbane, Australia; 2 Helmholtz-Institute for Functional Marine Biodiversity, University of Oldenburg, Oldenburg, Germany; 3 Alfred Wegener Institute, Helmholtz-Centre for Polar and Marine Research (AWI), Bremerhaven, Germany; 4 Australian Institute of Marine Science, Townsville, Australia; 5 Institute for Marine and Antarctic Studies, University of Tasmania, Hobart, TAS Australia; 6 School of Environment and Science, Griffith University, Nathan Campus, Brisbane, Nathan, QLD Australia; 7 Department of Biosciences, Swansea University, Swansea, SA2 8PP UK; 8 Commonwealth Scientific and Industrial Research Organisation, Brisbane, Australia

**Keywords:** Coral recruitment, Coral larvae, Microbial communities, Settlement inducer/inhibitor, 16S rRNA amplicon sequencing, Reef restoration

## Abstract

**Background:**

Crustose coralline algae (CCA) enhance coral recruitment, but the response of coral larval settlement to CCA varies between CCA species. Furthermore, it is unclear whether coral larvae respond to settlement cues from the algal host itself or its associated microorganisms. To determine whether CCA-derived settlement cues have a microbial origin, we interrogated the microbiome of 14 coralline algal species and a calcareous non-coralline alga eliciting varying levels of settlement across 14 coral species from a wide diversity of families found in the Great Barrier Reef.

**Results:**

Linear regression, differential abundance, indicator species, and random forest analyses were used to identify microbial taxa associated with high or low coral settlement. We found that the relative abundance of specific microbial amplicon sequence variants (ASVs) correlated with settlement and that these responses were largely coral species-specific. A select few microbial taxa associated with high or low settlement were shared across the corals *Dipsastrea favus*, *Echinophyllia aspera*,* Lobophyllia corymbosa*,* Mycedium elephantotus*, and *Platygrya sinensis*, suggesting potential shared settlement or inhibition cues. While shared ASVs associated with high coral settlement were found across multiple CCA species, low settlement ASVs were confined to few low settlement CCA species. *Candidatus Nitrosopumilus* and *Filomicrobium* microbes were found as potential shared microbial inducers, and members of *Pirellulaceae* and *Flavobacteriaceae* were identified as potential settlement inhibitors.

**Conclusions:**

These findings contribute to our growing knowledge of potential coral larval settlement cues and provide deeper insights into the link between the CCA microbiomes and coral recruitment.

**Supplementary Information:**

The online version contains supplementary material available at 10.1186/s40793-026-00907-6.

## Introduction

The growth of coral reefs worldwide crucially depends on successful reproduction and larval recruitment of single individuals. Ongoing severe climate-related events (e.g. elevated sea temperatures and subsequent coral bleaching) have negatively impacted corals’ ability to successfully reproduce and survive [1, 2]. The planktonic phase of coral larvae is especially sensitive to environmental disturbances, as larval development and swimming behaviour are markedly affected at higher sea water temperatures and lower pH levels [3, 4]. This further alters the ability of larvae to settle in response to environmental cues, metamorphose, and survive, thus creating a major bottleneck that limits new coral growth and genetic diversity on reefs [5]. In addition, different environmental settlement cues are likely simultaneously being altered in response to environmental changes (i.e., pH [6], water temperature [7], nutrient gradients [8], and proximity to anthropogenic pressure [9]). However, a comprehensive assessment of this is lacking as the specific cues that trigger coral larval settlement are largely uncharacterised.

The planktonic larvae of most coral species actively select a suitable settlement substrate [10, 11], with a variety of environmental cues including surface structures [12, 13], sound [14], colour [15–17], light [18], and biochemical cues [19, 20] influencing this selection. Many coral species have been shown to settle in response to crustose coralline algae (CCA), calcifying red algae that are abundant on tropical reefs and play a key role in maintaining coral reef structure and biodiversity [21–23]. Corals typically have a preference for specific CCA species [11, 24–30], and some studies suggest that corals may in fact settle in response to cues originating from CCA surface-associated microorganisms [28, 31] rather than the CCA themselves. Prior research has shown that CCA surface microbiomes are distinct from the surrounding water column [32, 33] and that microbial community composition varies between CCA species [23, 28, 34]. However, it has been challenging to untangle the effects of CCA microbial communities from the CCA host on coral larval settlement.

To separate host and microbial effects, analysis of crude extracts and microbial strains isolated from the CCA surface has uncovered morphogens that induce high levels of coral larval settlement [35, 36]. For example, two different strains of *Pseudoalteromonas* sp., isolated from the CCA species *Neogoniolithon fosliei* and *Porolithon* (*Hydrolithon*) *onkodes* [36, 37] produce the morphogen tetrabromopyrrole (TBP) that can induce larval settlement and metamorphosis in *Acropora* corals. More recently, cycloprodigiosin, an alkaloidal pigment produced by a *Pseudoalteromonas rubra* strain isolated by the CCA *Hydrolithon reinboldii*, was found to induce coral settlement once activated under high-light conditions [38]. However, the morphogenic attributes of any given microbe grown in cultivation might differ when part of a microbial community in the natural environment [39]. For example, *Pseudoalteromonas* were found to be less inductive when in mixed-species biofilms [40], while extracts of TBP are often unstable and its effect on settlement can be concentration and strain dependent [41].

Other studies have administered antibiotics to CCA surfaces to remove CCA surface microbial communities and isolate the inductive capacity of the CCA host on larvae of different marine invertebrates, with contrasting results [42–45]. Johnson and Sutton [42] found that removing microorganisms from CCA surfaces negatively impacted Crown-of-Thorns starfish larval settlement, but settlement response improved after re-inoculating single strains to CCA surfaces. Other studies found that coral larval metamorphosis response to CCA was not affected by the antibiotic treatment but still identified single strains (*Pseudoalteromonas*) that promoted larval settlement [43]. However, CCA surface microbiomes consist of diverse communities, and despite these advances, our understanding of the role that CCA-associated microbial communities play in promoting coral larval settlement for a wide diversity of coral species remains in its infancy.

Abdul Wahab et al. [11] explored larval settlement of 15 different Great Barrier Reef (GBR) coral species in response to 14 coralline algae species and the non-coralline alga *Ramicrusta*. They found that different CCA elicited varying coral larval settlement responses depending on the coral species [11]. After Abdul Wahab et al. [11] finished scoring the larval settlement, we additionally sampled each CCA microbiome. This experimental design provided the opportunity to further investigate whether these settlement differences were associated with specific CCA microbial communities. Hence, in this study we extend Abdul Wahab et al.’s findings to microbiome-related settlement cues by examining whether variations in microbiomes found on the CCA hosts used in their settlement experiment were associated with differences in larval settlement responses. Further, we aimed to identify taxa associated with high or low settlement and determine whether these taxa are coral species-specific or common across multiple species. Using a combination of statistical approaches, we identified individual microorganisms associated with high settlement for multiple coral species, and unique microbial taxa associated with low settlement inducing CCA species.

## Methods

Full details of the coral and algal collection and identification, spawning, maintenance of larval cultures, and settlement assays are described in Abdul Wahab et al. [11] and are summarised briefly below.

### Coral collection, spawning and larval culture

Colonies from fifteen different coral species, across 5 taxonomic families, from the Great Barrier Reef (GBR) were collected and used in settlement assays (*Acropora hyacinthus*,* A. tenuis*, *A. anthocercis*,* Caulastrea furcata*, *Coelastrea aspera*, *Dipsastrea favus*, *Echinophyllia aspera*, *Fungia fungites*, *Goniastrea favulus*, *Lobophyllia corymbosa*,* Montipora aequituberculata*, *Mycedium elephantotus*, *Platygyra daedalea*, *Platygrya sinensis*, and *Porites lobata*). Coral colonies were collected between the 9th to 20th October and 14th (Table S1) to 21st November 2021 (Table S2) from Magnetic Island (19˚07’45.78”S 146˚52’40.14”E), the Palm Island Group (18˚45’56.4”S 146˚32’2.58”E) and Davies Reef (18˚49’13.5”S 147˚38’40.32E) at 1–9 m depths under the GBRMPA Permit G21/45348.1. Corals were collected on SCUBA using a hammer and chisel and were transported into 70 L aquaria with consistent flow-through seawater for 4–6 h until their arrival at the National Sea Simulator (SeaSim) facility at the Australian Institute of Marine Science (AIMS) in Townsville, Australia. At SeaSim, corals were held in outdoor semi-recirculating aquaria with 1 μm filtered seawater at ~ 27.2˚C and natural light.

When the setting of gametes was observed, colonies that produce egg-sperm bundles were moved to separate aquaria and buoyant bundles collected within the first hour of their release and gametes were fertilised. Embryos were gently washed with filtered seawater (FSW) to remove extra sperm after one hour and transferred to either 500 L–70 L flow-through culture tanks. For *P. lobata*,* F. fungites*,* L. corymbosa* and *G. favulus* (i.e. gonochoric species, or hermaphroditic species releasing eggs and sperm separately), water containing sperm was mixed in aquaria that contained colonies with eggs, and embryos transferred to culture tanks within 30 to 45 min after signs of cleavage were observed. All larval cultures were maintained in flow-through culture tanks at ~ 27.2˚C until the settlement experiment.

### Algal collection and identification

Thirteen non-geniculate CCA (*Adeylithon* cf. *bosencei*, *Hydrolithon* cf. *reinboldii*, *Lithophyllum* cf. *insipidum*,* L.* cf. *kotschyanum*, *L.* cf. *pygmaeum*, *Lithothamnion* cf. *proliferum*, *Melyvonnea* cf. *madagascariensis*, *Neogoniolithon* cf. *fosliei*, *Porolithon onkodes*,* Porolithon* sp.1, *Porolithon* sp.2, *Sporolithon* sp., and *Titanoderma* cf. *tessellatum*), one geniculate coralline alga *Amphiroa* cf. *foliacea*, and one calcareous non-coralline alga *Ramicrusta* sp. (Peyssonneliaceae) were collected at 1–10 m depth on SCUBA by GD-P and MAW (Table S3). Each species was collected from a single patch habitat that had either low-, moderate- and high-light with a hammer and chisel from Davies Reef and Havannah Island between the 9th and 20th of October 2021 (Table S3). The 15 species are collectively hereafter referred to as CCA. Algal identification was first performed visually looking at anatomical and morphological traits and further identified with molecular analysis as previously detailed in Abdul Wahab et al. [11] and Diaz-Pulido et al. [46].

Collected CCA were moved to AIMS SeaSim and cut into 10 × 10 mm pieces with a wet diamond band saw (Gryphon) and glued on a poly-vinyl-chloride (PVC) rack. These racks were placed in indoor semi-recirculating aquaria with 1 μm filtered seawater (~ 3 turnovers per day) at ~ 27.2˚C and held for either 2 − 3 weeks (October spawning, Table S1) or 4 − 6 weeks (November spawning, Table S2). Aquaria tanks were cleaned twice weekly and herbivorous snails (*Caltholtia* and *Turbinaria*) were added to reduce macroalgal overgrowth. CCA were kept under light conditions resembling those of their collection sites and depths. The maximum midday irradiance levels for low-, moderate- and high-light adapted CCA was 12.7 − 15 µmol quanta m^− 2^ s^− 1^, 56 − 58 µmol quanta m^− 2^ s^− 1^, and approximately 120 µmol quanta m^− 2^ s^− 1^, respectively.

### Settlement assays and CCA sampling

**Fig. 1:**
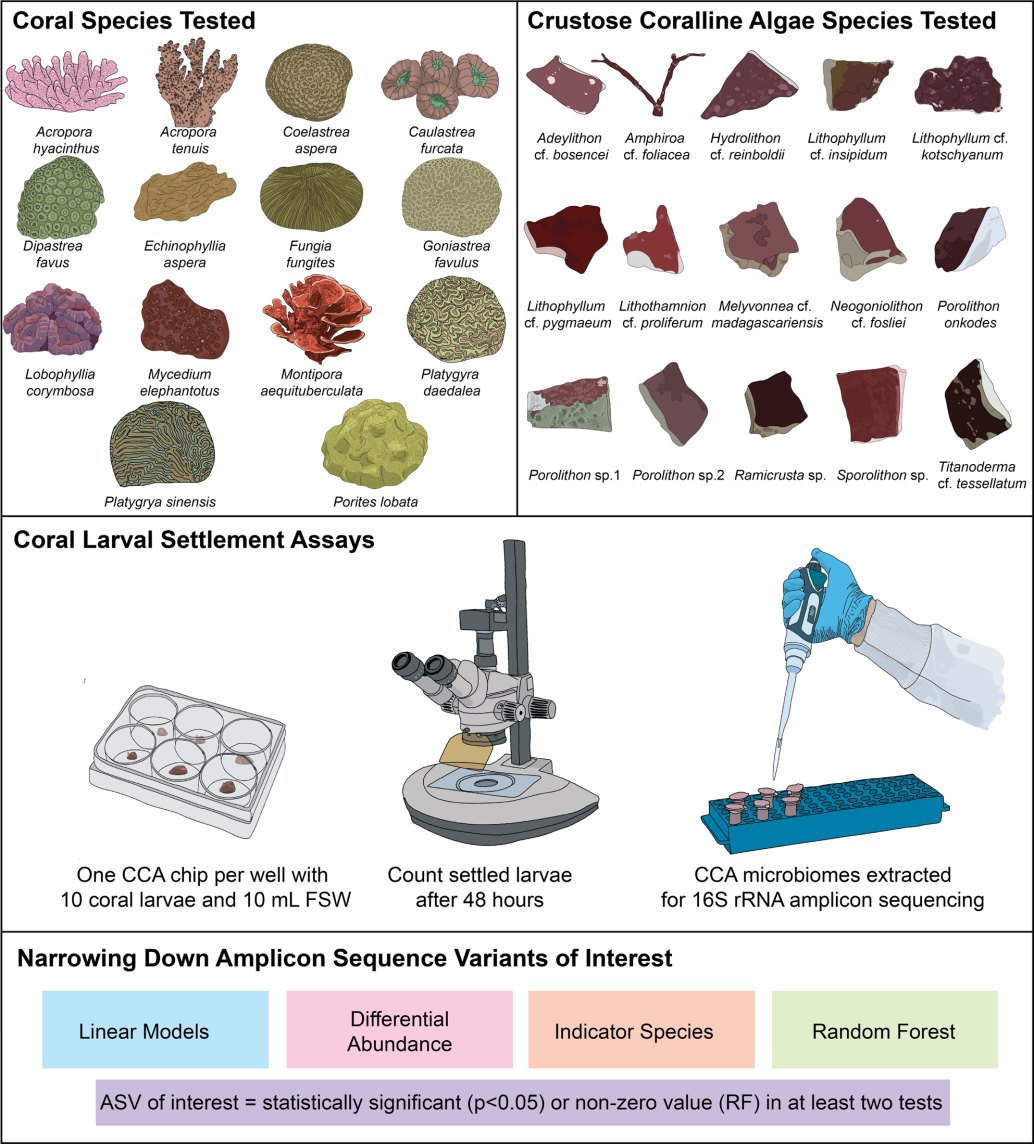
Understanding the role of CCA-associated microbial communities in coral larval settlement using an integrated approach. The diagram shows the three stages (coral and CCA species choice, settlement assays, ensemble of statistical analyses) used in this study to identify the key microbial drivers of coral larval settlement. Fourteen coral and 15 crustose coralline algae (CCA) species from the Great Barrier Reef were tested in coral larval settlement assays in Abdul Wahab et al. (2023). This study further characterized the microbiome of each CCA chip used in that experiment. Linear models, differential abundance, indicator species, and random forest analyses were used to narrow down amplicon sequence variants (ASVs) that were associated with high and low settlement for each coral species

Settlement assays were conducted in 6-well plates (Costar) with 10 mL of 0.1 μm FSW per well (Fig. 1). Ten active larvae within their competency period (~ 4–8 days; Table S1, S2) [47] and one 5 × 5 mm visually healthy CCA chip (tissue-side up) were placed in each well. Twelve replicates of each CCA treatment were randomised across 36 plates for each coral species except for *C. furcata*, where only 6 treatment replicates were used due to low larval stock. Autoclaved aragonite chips and blank wells were used as controls. Settlement was recorded after 46–55 h by counting permanently attached and metamorphosed larvae under a dissecting microscope. Settlement assays were repeated three times for *C. aspera* at different larval ages (5, 8 and 18 days) due to initial low competency at 5 days, with the 8-day larval settlement assay results analysed in this study. Further, samples from *A. anthocercis* were omitted from further analysis due to indiscriminate settlement of larvae (up to 40%) when CCA cues were not present (controls).

After settlement was recorded (Fig. 1; Table S4), each CCA chip (*n* = 12 replicates) was placed in a sterile cryovial and frozen in liquid nitrogen. Five of the twelve replicates were selected for CCA microbiome analysis. These replicates were chosen to cover a range of larval settlement outcomes, which allowed us to test if the variation in microbiome composition within a CCA species correlated with settlement success. The chip with the highest and lowest settlement response and three chips within the 50th percentile of settlement scores were chosen for sequencing (Table S4). FSW used in the settlement assays was sampled through the intake lines with 5 L per sample filtered onto 0.2 μm Sterivex filters (Millipore/Merck) for DNA extraction. Three replicate larval samples were also collected for each coral species to differentiate any larvae-associated microbes from the CCA microbiome. CCA, coral larvae, and water samples were stored in -75˚C freezers at AIMS until DNA extraction at the Australian Centre of Ecogenomics (ACE), University of Queensland, Brisbane.

### DNA extraction, sequencing and bioinformatic analysis

DNA was extracted from CCA samples (*n* = 75 per coral species, except for *C. furcata* due to lower assay replication [*n* = 45] and *C. aspera* [*n* = 71] where four samples were removed due to poor extraction results; Table S4) and blank extraction controls (a blank tube with no CCA chip present; *n* = 1 per coral species) following a lysozyme and proteinase K buffer extraction protocol described in Wilson et al. [48]. DNA was extracted from Sterivex filters containing FSW using the Phenol: Chloroform: IAA method described in Botté et al. [49] and pools of approximately 100 coral larvae were used for DNA extraction with the DNeasy^®^ UltraClean^®^ Microbial DNA extraction kit (Qiagen) following the manufacturer’s instructions. DNA quality was measured with a nanodrop (Thermo Scientific) for 260/280 and 260/230 absorbency ratios and quantified with a Qubit 1.0 Fluorometer and Qubit dsDNA HS assay kit (Invitrogen) before storage at -20˚C until sequencing.

Sequencing was conducted at ACE using 16 S rRNA gene amplicon sequencing targeting the V4 region on the Illumina MiSeq platform (2 × 250 bp) with primers 515 F ‘GTGYCAGCMGCCGCGGTAA’ and 806R ‘GGACTACNVGGGTWTCTAAT’ [50] with the Q5 Hot Start High-Fidelity 2X Master Mix (New England Biolabs) and the following PCR conditions: 98˚C 2 min, 25 cycles of 98˚C 10s, 55˚C 30s, and 72˚C 30s, and a 72˚C 2 min final extension. Demultiplexed sequences were processed in QIIME2 (version 2022.8) and denoised with the DADA2 plug-in [51], which merges pair-ends and maps reads into amplicon sequence variants (ASVs). The forward sequences were truncated to 245 bp, while the first 7 bp of the reverse sequences were removed to eliminate reduced quality bases and additionally truncated at 183 bp. The QIIME2 feature-classifier function fit-classifier-naive-bayes was used to train the classifier on the V4 region with the SILVA database (SSURef NR99 release 138.1) [52], which was further used with the function classify-sklearn to classify the ASVs. Samples with less than 3,000 reads in total were removed from further analyses. Specifically, two *C. aspera* samples (*n* = 69), two *P. daedalea* samples (*n* = 73), one *E. aspera* sample (*n* = 74), one *D. favus* sample (*n* = 74) and one *M. elephantotus* sample (*n* = 74) were removed. Read distribution per sample and rarefaction curves can be found in Table S4. ASVs classified as Eukaryote, mitochondria, and chloroplast were removed from the ASV table (< 0.03% of total reads). Relative abundance of ASVs was calculated per sample and ASVs with < 0.01% relative abundance in all samples were removed. This included all sequences found in blank extraction controls, filtered seawater and coral larvae samples.

### Statistical analyses

#### Microbial community composition

To identify microbial communities and specific microbial taxa associated with different larval settlement levels we performed community structure and differential abundance analyses of the microbiome [53].

Permutational multivariate analysis of variance (PERMANOVA) and pairwise PERMANOVAs were used to test whether microbial community composition differed between groupings of biologically relevant features. In particular, they were conducted between sample type (CCA, water, and coral larvae species), CCA conditioning month (October vs. November), and between CCA species to determine whether CCA microbiomes were distinct from those found in surrounding seawater, coral larvae, and between CCA species. For each coral species, pairwise PERMANOVAs were performed to compare CCA microbial communities associated with different settlement categories (low [0–30%], medium [30–60%] and high [60–100%], defined through histogram partitions as described in Turnlund et al. [54]), and to ascertain if microbial community composition differed according to the associated settlement response (Fig. S1). All PERMANOVAs were performed on distance matrices created from log(x + 1) transformed ASV counts. The function vegdist() was used to calculate Bray-Curtis dissimilarity matrices, adonis2() for PERMANOVAs, and pairwise.adonis() for pairwise PERMANOVAs from the vegan package (version 2.6.4) [55]. P-values were adjusted with the Benjamini-Yekutieli (‘BY’) method [56]. All statistical analyses were performed in R (version 2023.6.0.421) [57].

Microbial communities from CCA, coral larvae and seawater were compared using non-metric multidimensional scaling (nMDS) ordination plots based on Bray-Curtis dissimilarity. Similarly, for each coral species, CCA microbial communities were compared between different CCA species and larval settlement scores. ASV count matrices were log(x + 1) transformed before creating distance matrices with the metaMDS() function from the vegan R package [55]. All plots were visualised with ggplot2 (version 3.4.4) [58].

#### Identifying ASVs associated with high or low settlement

A combination of complementary statistical analyses, including linear models (LM), differential abundance, and indicator species, and machine learning analyses were used to identify specific microbial taxa associated with settlement. Indicator species analysis evaluates the strength of an ASV association with metadata variables based on both presence and relative abundance. We performed this analysis on the relative abundance of the filtered ASV table using the Multipatt() function of the Indicspecies package (version 1.7.14) [59]. Indicator analyses were computed at *p* < 0.05 significance, and alpha specificity and beta-fidelity values of 0.75 to identify indicator taxa for each settlement category per coral species. Indicator species values consider both site fidelity and specificity scores to assign a single indicator score between 1 (an ASV that is found in every sample within a specific settlement level and only that specific settlement level) and 0 (an ASV that is not exclusive or persistent amongst samples in a certain settlement level).

Differential abundance was calculated using the ancombc2() function from the ANCOM-BC package (version 2.0.3) [60, 61], which models the microbiome data with a linear regression framework in log-scale and reports significant log fold changes (LFC) between sample groups. The p-value was adjusted with the false discovery rate method (FDR) method, taxon proportion filters were set to zero (prv_cut = 0) since data was pre-filtered, and the parameter “Pairwise” was set to TRUE to account for pairwise comparisons between all settlement levels. Significant log-fold changes at the ASV level between high and low settlement-inducing CCA species were visualised for each coral assay with a bar plot using the package ggplot2 [58].

Correlations between ASV abundance and coral settlement values were also analysed using multivariate linear models (LM) with the R package Maaslin2 (version 1.12) [62]. Models were run for each coral separately and microbial data (post-filtering at 0.01% minimum abundance and 0.1% minimum prevalence) was log-transformed and normalised with total-sum scaling. CCA host was considered a random effect, and the continuous percent settlement score was used as a fixed effect.

A Random Forest (RF) analysis using Breiman’s random forest algorithm was conducted for each coral to identify ASVs with the highest predictive ability for coral settlement with the randomForest package (version 4.7.1.1) [63, 64]. RF models were run with 500 trees and considering percent settlement as the response variable (Table S5). ASVs with non-zero IncNodePurity variable importance values were kept if the ASV also had significant results in LM, indicator species and/or differential abundance analyses.

#### Data visualisation

Coral larval settlement scores per CCA treatment were visualised with box plots in ggplot2 [58] for each coral. For each individual coral species, ASVs were further narrowed down based on whether they were found to be significantly correlated with settlement (*p* < 0.05) for at least two of the following: LM analysis, differentially abundant (ANCOM-BC), an indicator for high/low settlement (Indicspecies) and/or non-zero RF model importance values (Fig. 1). LM coefficient score, log fold change, indicator, and RF model importance values for each ASV of interest were visualised with bar plots and the relative abundances of the ASVs across CCA samples were presented using bubble plots in ggplot2 [58]. CCA samples that did not contain any of the ASVs of interest were removed from the bubble plot. For visual comparisons across coral species, the significant Maaslin2 LM results were shown in heatmaps using pheatmap (version 1.0.12) [65] and ggplot bar charts [58].

## Results and Discussion

**Fig. 2:**
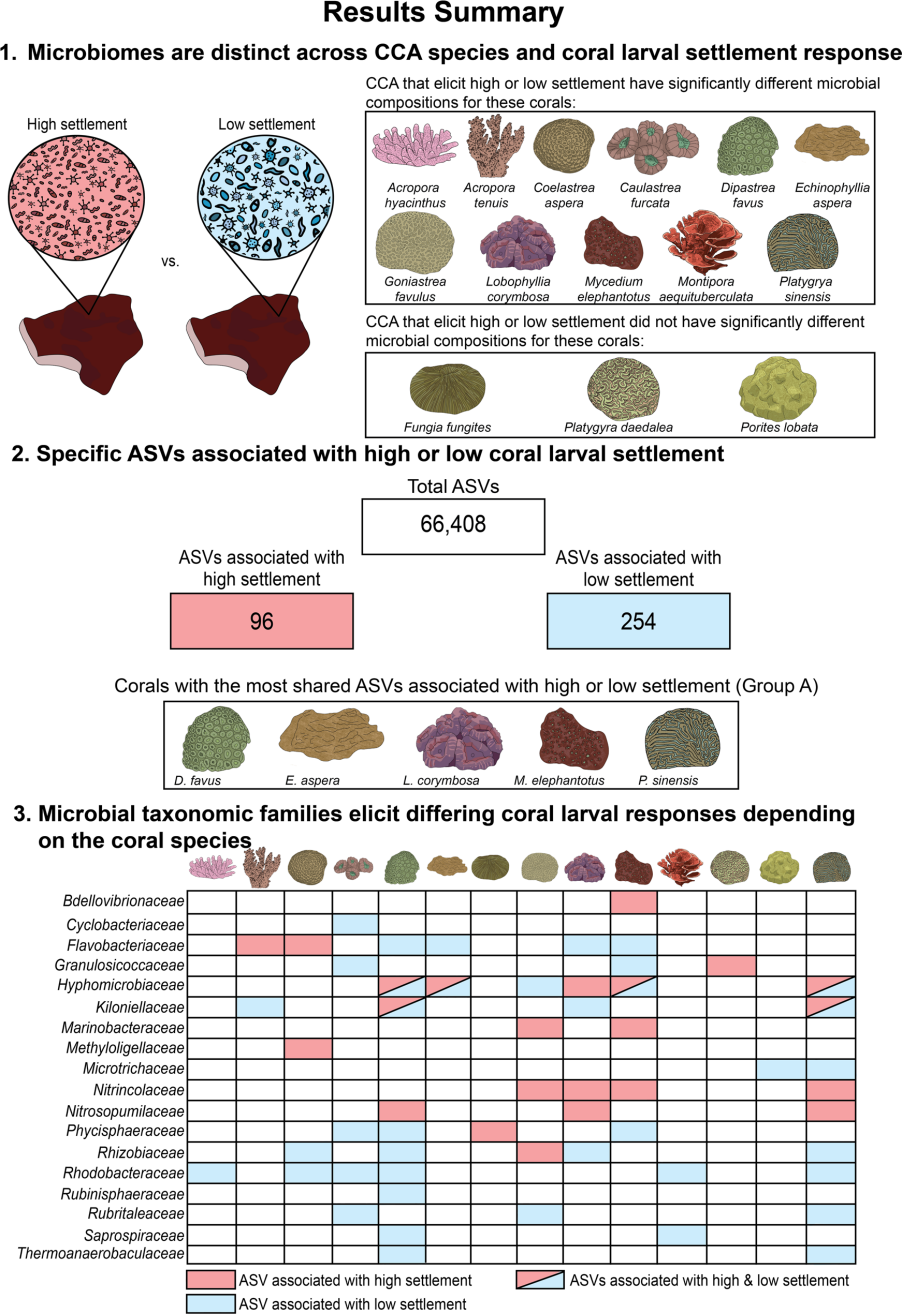
Assessing ASVs associated with high or low coral settlement resulted in a specific consortium of ASVs per coral species. This diagram summarises the overarching results of this study including (1) the groups of corals where CCA microbial communities that were associated with high or low coral larval settlement were significantly distinct, (2) the total number of ASVs associated with high or low settlement, and (3) the ASV microbial taxonomic families associated with high and low settlement across all 14 coral species

To investigate the potential role of crustose coralline algae (CCA) microorganisms in coral larval settlement, this study interrogated the microbiomes of 15 CCA species that elicited varying settlement responses for 14 coral species commonly found on the Great Barrier Reef (GBR). We found that although CCA microbiomes were distinct and species-specific, there was also minor microbial compositional variation within CCA species. Importantly, differences within CCA species microbiomes reflected differences in coral larval settlement responses (Fig. 2). We identified individual microbial taxa that correlated with high or low settlement as candidate inducers or inhibitors.

### Microbiomes are distinct across CCA species and coral larval settlement response

**Fig. 3:**
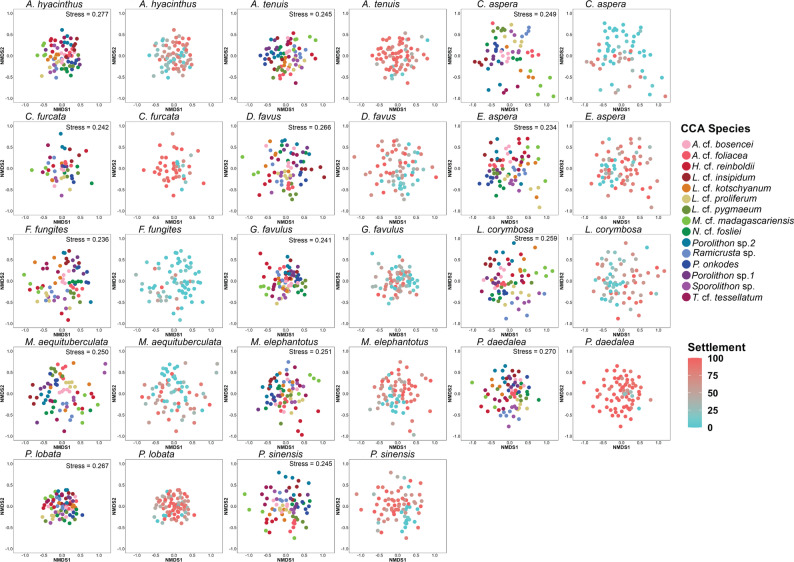
Microbial communities differentiate by crustose coralline algae (CCA) species and by coral larval settlement strength. Non-metric multidimensional scaling (nMDS) ordination plots based on Bray-Curtis dissimilarity comparing microbiome composition for CCA samples collected per coral species settlement assay. For each coral, two similar nMDS plots are shown, one coloured by CCA species (left) and one coloured by larval settlement score (right)

**Fig. 4:**
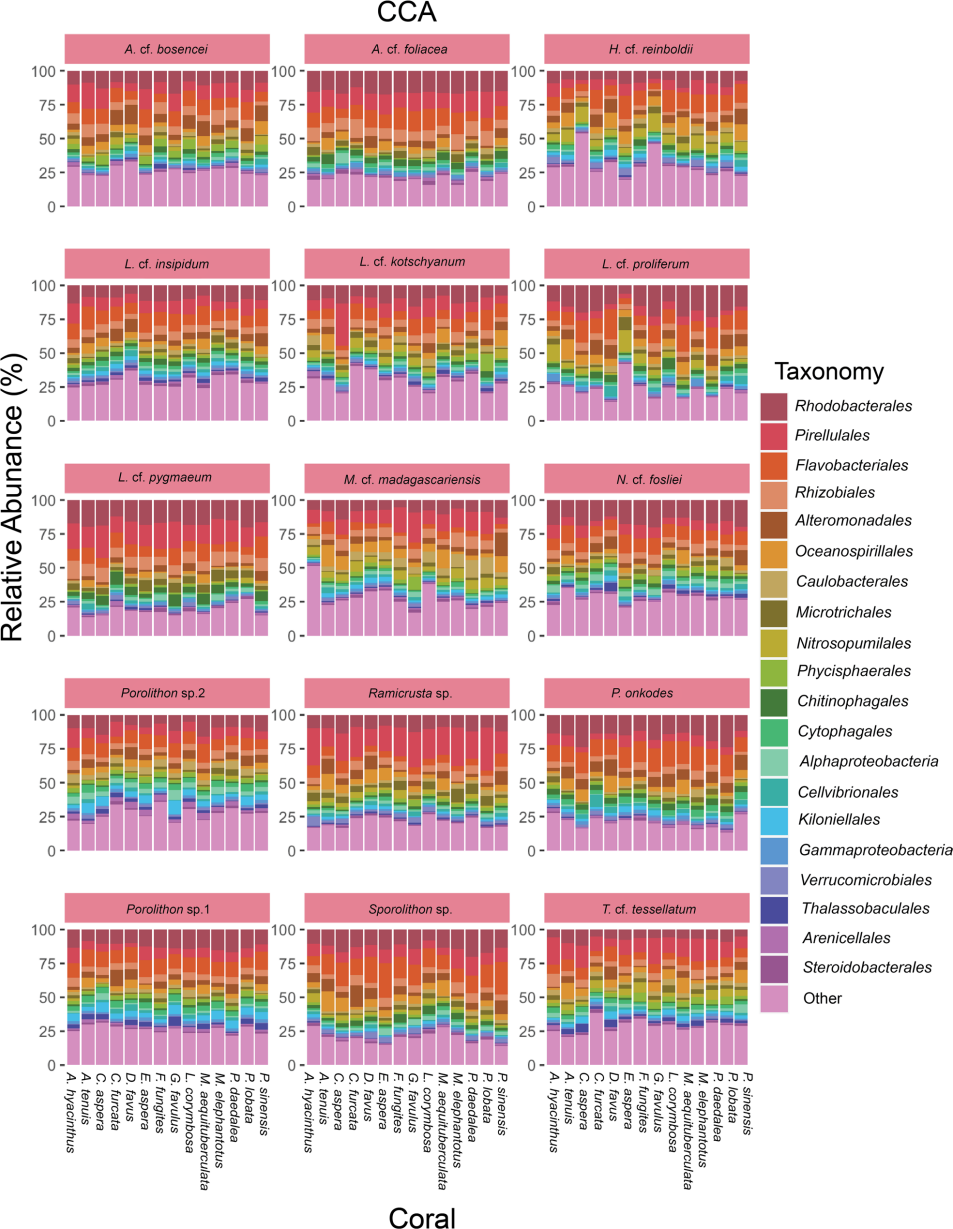
Microbial community composition varies across crustose coralline algae (CCA) species. Each bar shows the mean relative abundance per CCA species sampled for each coral species’ larval settlement experiment. The top 20 taxonomic orders are shown, and the less abundant orders were combined in the ‘Other’ category

CCA microbial communities were distinct from the surrounding seawater and coral larvae (PERMANOVA: F = 7.16, *p* < 0.001; Table S6; Figure S2), and they also differed between CCA species at the amplicon sequence variant (ASV) level. Previous research has shown that CCA host species-specific microbial communities [28, 31, 34], and our results highlight that this pattern persists in aquaria after collection from the field (Fig. 3; see Table S7 for full PERMANOVA results). We note that CCA microbial communities may shift once moved to aquaria and we observed shifts in composition between assay months (PERMANOVA: F = 7.30, *p* < 0.001; Table S8). However, the microbial communities eliciting high or low coral larval settlement were still distinct. CCA species shared similar microbial orders, such as *Rhodobacterales*,* Flavobacteriales*,* Pirellulales*,* Rhizobiales*, and *Altermonadales*, consistent with previous reports of CCA microbiomes [28, 31, 34, 66] (Fig. 4).

**Fig. 5:**
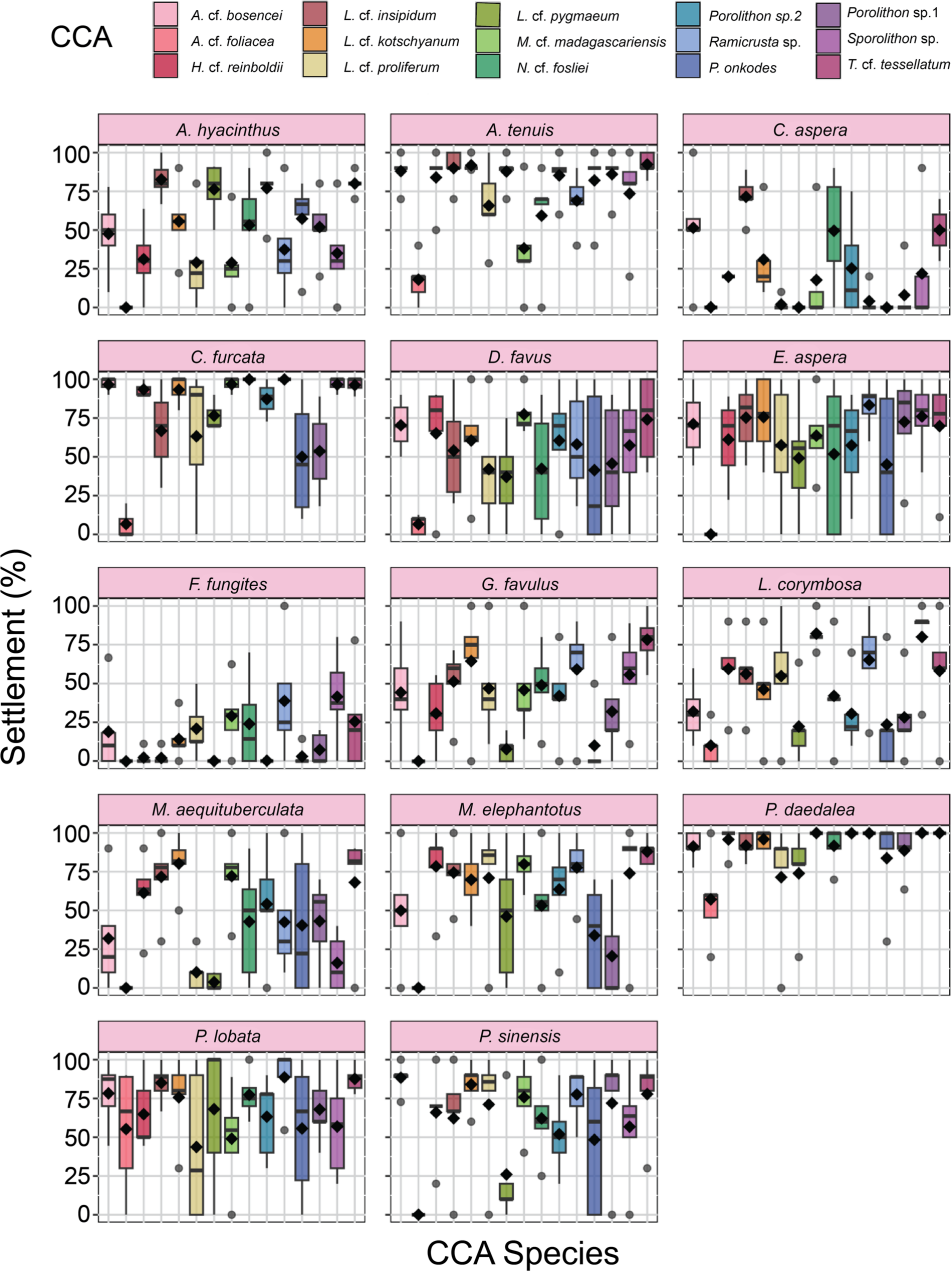
Coral larval settlement preferences differ by coral species and vary within crustose coralline algae (CCA) species. Each panel shows the results for a single coral species and box plots are coloured by CCA species. The results presented here are a subset of the samples from Abdul Wahab et al. [11] and were selected across the range of settlement values for each CCA and coral combination (*n* = 5 per CCA for each coral species, except for corals with dropped samples as specified in the methods). Each diamond represents the mean settlement for the samples sequenced for this study (see Abdul Wahab et al. [11] for full CCA settlement response data)

Settlement responses of coral larvae varied both across and within CCA species (Fig. 5) [11]. We further investigated whether this variation was driven by CCA microbial composition for each coral species. CCA microbial communities mostly grouped according to CCA species, with some CCA species and some samples within CCA species promoting higher settlement than others (Fig. 3; see Table S7 for full PERMANOVA results). For example, when comparing high and low settlement groups across CCA species, there was a significant difference between microbial communities associated with high versus low settlement for every coral tested, except for *F. fungites*,* P. daedalea*, and *P. lobata* (Table S9). It is still unclear whether coral larval settlement cues originate from the CCA host, their epiphytic microbial communities, or a combination of both. However, since CCA associated with low settlement had significantly different microbial communities than CCA associated with high settlement, there may be a microbial settlement cue for most of the corals tested here. Where no significant difference was observed between inductive and non-inductive CCA microbiomes, abiotic or host derived cues may explain the patterns found in coral settlement instead (*F. fungites*,* P. daedalea*, and *P. lobata*; Table S9).

Gómez-Lemos et al. [67] found that *Titanoderma* cf. *tessellatum* chemistry had a stronger effect on *Acropora millepora* settlement than *T. tessellatum* surface microbial communities and the microbial communities were only inductive in the presence of algal dissolved organic carbon (DOC). In our study, *T.* cf. *tessellatum* elicited differing settlement responses per coral species (Fig. 5). Interestingly, for some corals (i.e. *C. aspera*,* E. aspera*,* L. corymbosa*, and *M. aequituberculata*; Fig. 3), there was a significant difference between microbial communities associated with high and low settlement, suggesting potential involvement of the microbiome. On the contrary, such differences in microbiome composition were not observed for *A. millepora* settlement, aligning with the findings of Gómez-Lemos et al. [67]. Meanwhile, Giorgi et al. [45] administered a range of antibiotics to CCA surfaces to differentiate host and microbial cue responses for *Orbicella faveolata*, and found that larval settlement increased with antibiotic treatment for some CCA species (likely a host originated cue paired with the decrease of a microbial inhibitor), but decreased settlement for other CCA species (likely a microbial cue). Therefore, the origin of these cues (host vs. microbial vs. a combination of both) are likely coral species specific and cannot be generalised [45, 68]. While our findings suggest involvement of the CCA microbiome for some coral species based on differences in overall community composition, additional experiments are required to fully untangle the contributions of microorganisms, CCA host, and chemicals produced.

### Specific ASVs associate with high or low coral larval settlement

**Fig. 6:**
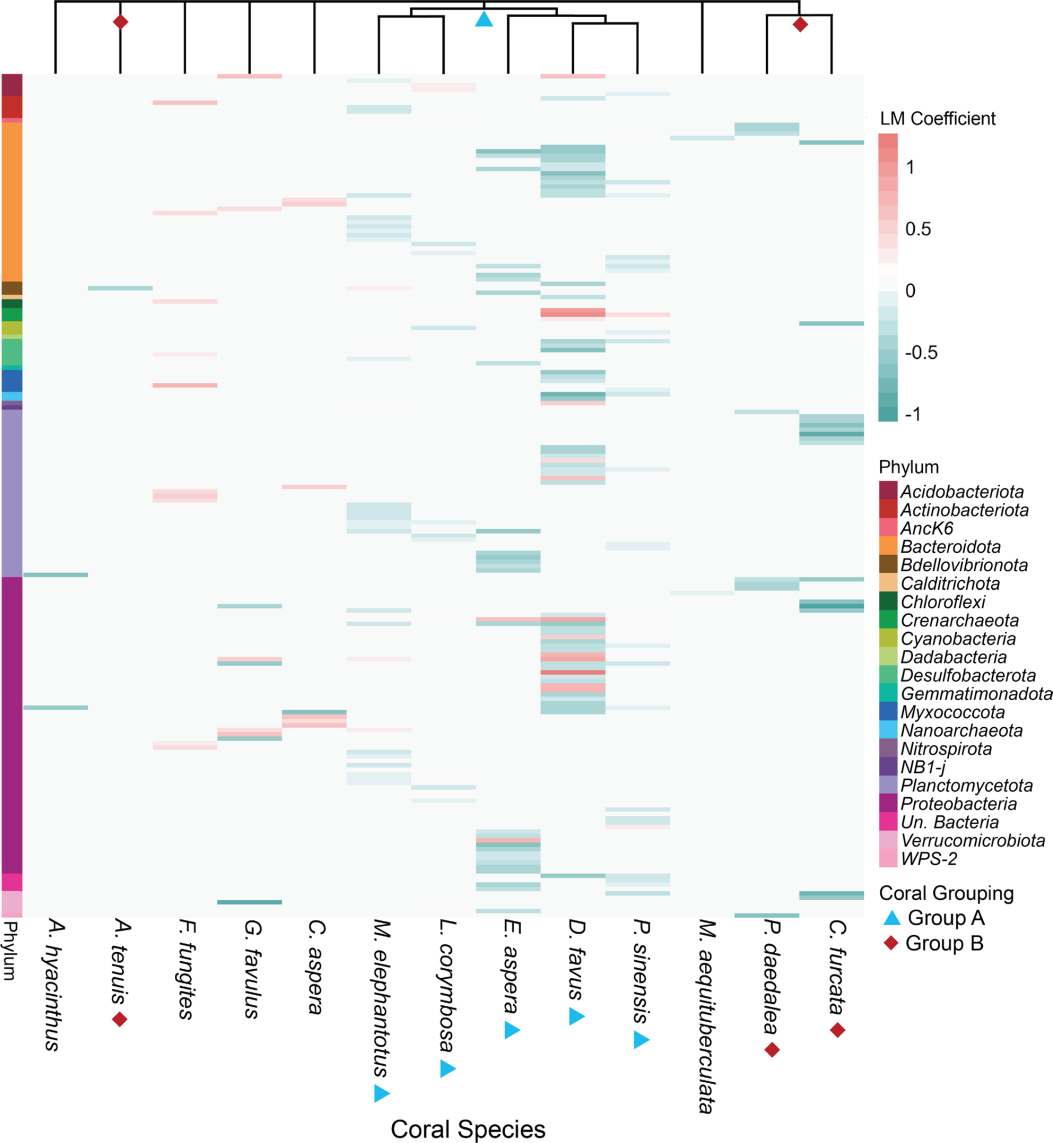
Amplicon sequence variants (ASVs) associated with high or low settlement for each coral species. Each row represents an ASV with a significant linear model regression coefficient (LM) and is coloured by this coefficient (Red = high settlement; Blue = low settlement). The dendrogram is clustered by corals that share similar ASVs associated with positive and negative linear regression scores. ASVs were chosen if they were significant in the linear model regression and at least one other test, including differential abundance, or indicator species or have a non-zero random forest value. Overall, 96 ASVs were associated with high settlement and 254 ASVs were associated with low settlement. ASVs are represented by rows and coloured on the left axis by the Phylum or the highest classification the ASV belongs to (labeled unassigned (un.)). Columns represent coral species with two groups of corals (Group A and Group B) showing similar CCA settlement preferences as identified by Abdul Wahab et al. [11]. *Porites lobata* (Group B coral) is omitted from this graph because no significant ASVs were identified to correlate with high or low settlement in the linear regression analysis

Multiple metrics were used to identify ASVs associated with high or low coral settlement, including linear models (LM), differential abundance (DA), indicator species analysis (IA), and random forests (RF). All analyses were performed at the ASV level, with ASVs taxonomically classified to the lowest possible resolution. ASVs were considered associated with a specific coral larval settlement level if they were identified by at least two of the LM, DA, IA, and/or RF metrics for higher confidence. This allowed us to narrow down taxa associated with high or low settlement and compare their presence and relative abundance across CCA species. After filtering out Eukaryote, mitochondria, and chloroplast reads (0.0003%, 0.008%, and 0.018% of the total reads, respectively), 66,408 unique ASVs were present in the dataset. ASVs significant in at least two of LM, DA, IA, and/or have non-zero RF values further identified 96 ASVs correlated with high coral settlement and 254 ASVs correlated with low settlement (Fig. 2; Table S10). Specifically, we identified a group of coral species that displayed similar groups of ASVs correlated with high or low coral settlement (*M. elephantotus*,* L. corymbosa*,* E. aspera*,* D. favus*, and *P. sinensis*: herein referred to as ‘Group A corals’ as first defined by Abdul Wahab et al. [11]) (Figs. 2 and 6). These corals were previously identified to have similar selective CCA settlement preferences [11], while a second group identified by Abdul Wahab et al. [11] (*A. tenuis*, *P. daedalea*,* C. furcata*, and *P. lobata*: herein referred to as ‘Group B corals’) did not share similar ASVs correlated with high or low settlement (Fig. 6).

**Fig. 7:**
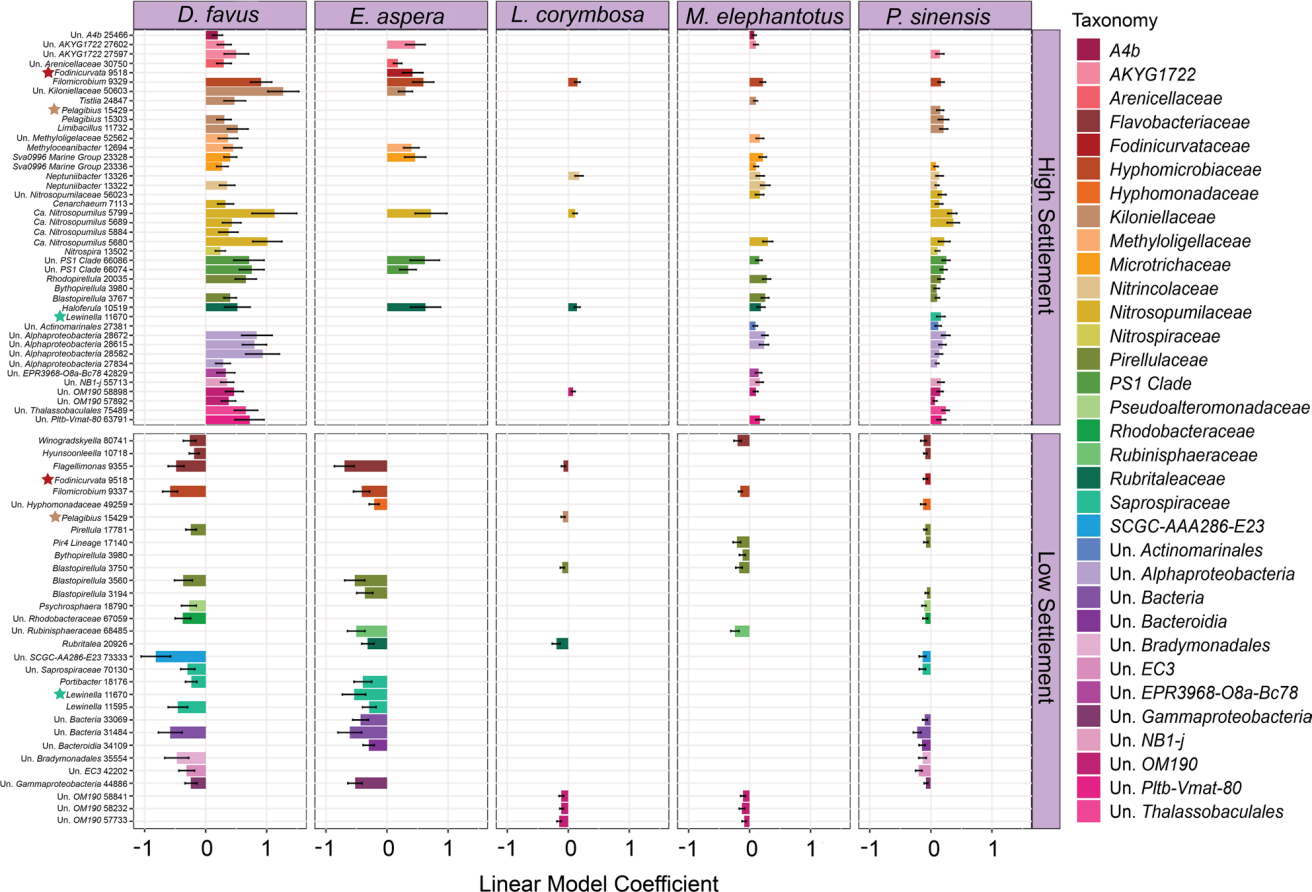
Amplicon sequence variants (ASVs) associated with high or low settlement that are shared amongst Group A coral species (*Dipsastrea favus*,* Echinophyllia aspera*,* Lobophyllia corymbosa*,* Mycedium elephantotus*,* and Platygrya sinensis*). Bar lengths represent the linear model coefficient value for ASVs that were significant for more than two species within coral Group A. Individual rows are labeled by the ASV genus name and a unique ASV identifier used in this study. Bars are coloured by the taxonomic family they belong to (left legend) and are separated by their associations with high or low settlement. ASVs with stars are associated with high settlement for one coral but associated with low settlement for another coral

For generalist coral larvae (i.e., larvae that do not show a strong preference for the CCA species they settle on), like Group B corals and to a lesser extent *Acropora hyacinthus*, fewer settlement-associated ASVs were identified compared to corals with selective settlement preferences (i.e., larvae that only settle on a few specific CCA species), like Group A corals (Fig. 6). Specifically, we found that Group A corals shared multiple common ASVs associated with high or low settlement, but only three ASVs from genera *Fodinicurvata*, *Pegibius* and *Lewinella* elicited different responses across different species of corals (e.g. *Fodinicurvata* was associated with high *E. aspera*, but low *P. sinensis* settlement; *Pegibius* was associated with high *P. sinensis*, but low *L. corymbose* settlement; *Lewinella* was associated with high *P. sinensis*, but low *E. aspera* settlement; Fig. 7). This coral species specificity at the ASV level highlights the importance of identifying potential inductive microbial taxa at the lowest taxonomic level possible. Notably, these associations were less clear at higher taxonomic levels, whereby taxonomic families comprised both inducing and inhibiting ASVs (Fig. 7). For coral species not included in Groups A or B, few or no ASVs were associated with high settlement (see Figure S3-16) suggesting their larvae selectively respond to CCA host derived cues, cues from other symbionts, or inhibitory cues.

### Shared taxa correlated with high settlement

Amongst Group A corals, *D. favus* and *P. sinensis* shared the highest number of ASVs associated with high settlement, while *L. corymbosa* had the lowest overlap (Fig. 7). In contrast, a study testing the settlement responses of similar coral species (*D. favus*, *E. aspera*, *L. corymbosa*, *P. lobata*, and *P. sinensis*) to microbial biofilms found little overlap in ASVs correlating with high settlement across corals species [69]. This may reflect differences in habitat niches between CCA and abiotic substrates, whereby inductive microorganisms may depend on resources provided by the CCA host to successfully colonise, enhanced cue potency in the presence of algal DOC [67], and/or direct larval response to the CCA host.

Despite the numerous ASVs associated with high settlement shared amongst the Group A corals, only one ASV assigned to *Filomicrobium* was shared across all (Fig. 7). This *Filomicrobium* ASV was associated with high settlement from LM analysis for each coral and was additionally found to have higher differential abundance (DA) in high compared to low settlement CCA for *D. favus* (Log Fold Change (LFC): 2.10 ± 0.44), *E. aspera* (LFC: 2.03 ± 0.54), and *P. sinensis* (LFC: 2.10 ± 0.44). *Filomicrobium* has been reported in healthy *Galaxea fascicularis* and *Porites pukoensis* corals [70], brown algae *Cystoseira compressa* [71], and green seaweed *Halimeda opuntia* [72] and was found in multiple CCA species in this study. Therefore, it may be indicative of healthy algae (free of disease), which could provide more inductive compounds than diseased algae.

The other Group A coral high settlement-associated ASVs were shared across a combination of coral species within Group A and not solely concentrated on specific high settlement inducing CCA, like *T.* cf. *tessellatum*. (Fig. 7). For example, one *Candidatus Nitrosopumilus* ASV was shared amongst all Group A corals except *L. corymbosa* for LM analysis and was also a high settlement indicator for *D. favus* (Indicator species value (ISV): 0.57) and *P. sinensis* settlement (ISV: 0.69) (Table S10). A second *Ca. Nitrosopumilus* was shared amongst *D. favus*, *M. elephantotus*, and *P. sinensis* for LM analysis, and showed significantly higher relative abundance in high settlement *D. favus* (LFC: 1.43 ± 0.48) and *P. sinensis* (LFC: 1.43 ± 0.48) samples (Table S10). *Nitrosopumilus* is an ammonia-oxidizing bacteria commonly found in sponges [73–75]. Certain members have been shown to produce nitric oxide [76], a key signalling molecule for marine invertebrate larval settlement [77–79]. Future research should explore the role of nitric oxide by *Nitrosopumilus* sp. in regulating coral larval settlement.

In addition, two *Neptuniibacter* ASVs also correlated with high settlement in Group A corals; one associated with *G. favulus* (LFC: 0.44 ± 0.12), *M. elephantotus* (LFC: 0.26 ± 0.08), and *P. sinensis* (LFC 0.10 ± 0.03) and the other with *L. corymbosa* (LFC: 0.18 ± 0.07) and *M. elephantotus* (LFC: 0.17 ± 0.07) (Table S10). This genus is known to occur in the microbiomes of the CCA species *P. onkodes* [6, 31], green algae [80], and *Mussisimilia* corals [81] and has previously been linked to coral settlement in *Acropora millepora* [31] and *Pocillopora damicornis* [82]. Here, high settlement *Neptuniibacter* ASVs were present in every CCA species tested, including low abundances in *Amphiroa foliacea*, which elicited little to no settlement from any coral (Figures S3-S16). Therefore, any settlement response initiated by *Neptuniibacter* is likely concentration dependent, influenced by other members of the biofilm community and/or coupled to a host cues. Overall, high settlement ASVs shared amongst Group A corals were found across multiple CCA species at varying relative abundances (see Figure S3-16) and these associated were consistent despite shifts in CCA communities between assay months (Table S8). These results suggest the corals tested here respond to microbial signals present across the different CCA species, yet further testing is required to confirm this hypothesis.

### Shared taxa correlated with low settlement

While no low settlement ASVs were shared across all five Group A corals, ASVs belonging to *Planctomycetes*, like *Pir4* and *OM190* lineages, and *Flavobacteriacae* were shared across a selection of Group A corals (Table S10). Microbes belonging to these groups are commonly associated with different types of algae (e.g., red [83], brown [84, 85], and green [86]) and have been shown to degrade polysaccharides in algal cell walls [86, 87]. Within Group A corals, *D. favus* and *E. aspera* shared the greatest number of ASVs associated with low settlement (Fig. 7). For example, a *Flavobacteriaceae* ASV belonging to the genus *Winogradskeylla* was correlated with low settlement for three of the species: *D. favus* (LFC: -0.98 ± 0.31), *M. elephantotus* (LFC: -1.40 ± 0.40), and *P. sinensis* (LFC: -1.0 ± 0.31) (Fig. 7; Table S10). *Winogradskeylla* microbes were found to be correlated with low *P. sinensis* settlement from microbial biofilms, suggesting that even separated from the CCA host, this microorganism may inhibit *P. sinensis* settlement [69].

Within the phylum *Planctomycetes*, ASVs belonging to the *Pir4* and *OM190* lineages and the genus *Blastopirellula* were correlated with low settlement in group A corals, although the specific ASVs varied among species (Fig. 7). For example, low settlement in *M. elephantotus* and *P. sinensis* was associated with a *Pir4* lineage ASV, while three *OM190* ASVs were correlated with low settlement in *L. corymbosa* and *M. elephantotus* (Fig. 7). Furthermore, the genus *Blastopirellula* was associated with low settlement across all five corals; with three *Blastopirellula* ASVs each linked to low settlement in a different pair of coral species: *L. corymbosa* and *M. elephantotus*, *D. favus* and *E. aspera*, and *E. aspera* and *P. sinensis* (Fig. 7). In contrast, *Pir4* lineage and *Blastopirellula* were associated with high *P. sinensis* and *P. lobata* settlement in response to microbial biofilms [69], suggesting that the settlement effect of these microorganisms may differ when paired with the CCA host. For example, the CCA host might be counteracting the settlement inducing ability of the microbes resulting in different coral larval settlement responses.

Although several taxa from the phylum *Planctomycetes*, such as Pir4 lineage and *Blastopirellula*, and the family *Flavobacteriaceae*, like *Winogradskeylla*, correlated with low coral settlement, these ASVs were unique to CCA with overall low settlement (i.e., *A.* cf. *foliacea*). This shows that CCA species with low coral settlement response often host microbial taxa that are not found in settlement-inducing CCA. Biocidals produced by some members of the phylum *Planctomycetes* and genus *Winogradskeylla* could directly curb colonisation of settlement-inducing microbes and/or shape the microbial community to deter larval settlement. For example, members of *Winogradskeylla* were previously identified in diseased coral [88] and alga microbiomes [83, 86, 89] and have reported biocidal activity by producing poly-ethers that inhibited barnacle *Balanus amphitrite* [90, 91] and *Hydroides elegans* settlement [92]. Similarly, *Planctomycetes* lineages are commonly found within algal microbiomes [84–86, 93, 94] and members of the phylum *Planctomycetota* have been associated with algal dysbiosis [87], degradation [95], and antibiotic resistance [87, 96–99].

Furthermore, CCA with unique low settlement-associated ASVs also often occupied mid-high light habitats, i.e., *A.* cf. *foliacea*. Abdul Wahab et al. [11] suggested that corals adapted to certain light habitats settled preferentially on CCA from similar light habitats, with Group A coral preferring CCA from mid-low light habitats. This preference could be mediated by microbes found on different CCA, as settlement of Group A corals largely negatively correlated with ASVs unique to high-light habitat CCA (Table S3; Figures S7-S8, S11, S13, S15) and CCA metabolomes for the species tested here may also change with light condition [46]. For example, low settlement ASVs were frequently found in low settlement samples of CCA found in high-light conditions (i.e., *P. onkodes*, *L.* cf. *pygmaeum* and *A.* cf. *foliacea*). *A.* cf. *foliacea* is a branched, geniculate coralline alga that induces weak settlement responses for a wide diversity of GBR coral species [11], despite the presence of microbial taxa that correlate with high settlement. While this could be attributed to the presence of microbial inhibitors, it may also be attributed to inhibitory signals from the host itself. Further research remains warranted to establish whether the apparent inhibitory effect is due to a specific microbial cue or the CCA host.

Most corals in this study had more ASVs correlated with low than high larval settlement levels and these were shared across a variety of CCA species. This trend is largely consistent with previous coral larval settlement studies [8, 54, 69, 82]. For example, *Lewinella* (*Saprospiraceae*) was correlated with low *M. aequituberculata* and *D. favus* settlement (Table S10) in this study and was also negatively correlated with *A. tenuis* coral settlement [8]. In addition, members of *Planctomycetes* and Rhizobiaceae were associated with low *C. furcata*,* M. elephantotus*,* D. favus*,* C. aspera*,* L. corymbosa* and *P. sinensis* settlement (Table S10) and were also found to correlate with low *A. tenuis* [8] and *P. damicornis* coral settlement [82], respectively. It has been postulated that *Rhizobiaceae* encourages macroalgae growth through enriched nitrogen fixation [82, 100]. *Rhizobiaceae* has also been found associated with coral disease [101–103] and bleaching [104], and has been assumed to be detrimental to larval settlement [82].

### Microbial taxonomic families elicit differing coral larval responses depending on the coral species

Within a single microbial family, some ASVs were associated with high settlement while others were associated with low settlement, depending on the coral species, further highlighting the complexity of settlement responses (Fig. 2). For example, one *Tenacibaculum* (Flavobacteriaceae) ASV was associated with high settlement of *A. tenuis* (ISV: 0.793) and was found in every single CCA species tested, with higher relative abundance in *L. insipidum* (mean relative abundance (MRA): 0.91 ± 0.7%) and *Porolithon* sp.2 (MRA: 1.33 ± 0.5%) samples (Figure S3). *Tenacibaculum* has previously been postulated to influence larval settlement of the coral *P. damicornis* [82] and the health of *Tubastraea coccinea* [105]. However, this same ASV was also associated with low *D. favus* settlement (LMC: -0.61 ± 0.22). *Tenacibaculum* and one unassigned *Flavobacteriaceae* were the only *Flavobacteriaceae* ASVs correlated with high settlement (for *A. tenuis* and *C. aspera*, respectively; Table S10), while all other significant *Flavobacteriaceae* ASVs identified (*Aquimarina*,* Flagellimonas*, and *Maribacter*) correlated with low settlement (Table S10). *Flavobacteriaceae* have also been associated with low settlement of mussels [106] and high settlement of the corals *A. microphthalma* [107], *P. sinensis*,* E. aspera*, and *P. lobata* [69]. Similar results were found for a *Granulosicoccus* ASV (*Granulosicoccaceae*), which correlated with high *P. daedalea* settlement (LM Coefficient: 0.35 0.11), but low *C. furcata* (LMC: -0.58 ± 0.10; LFC: -1.78 ± 0.45; ISV: 0.755) and *M. elephantotus* (LFC: -1.64 ± 0.4; ISV: 0.757) settlement. *Granulosicoccus* have previously been found in biofilms that elicited high larval settlement in coral *A. tenuis* [54], but a mixed settlement response between *P. daedalea*,* C. furcata*, and *M. elephantotus* (corals belonging to the *Merulinidae* family), suggesting that microbial cues vary within coral families, are species-specific, and that microbial taxonomy at the family and even genus level does not necessarily reveal the inductive capacity of a microbe.

*Rhodobacteraceae* is another taxonomic family previously associated with both high and low levels of coral larval settlement [8, 54, 69]. In this study, ASVs belonging to *Rhodobacteraceae* were correlated with low settlement in *C. furcata*,* M. aequituberculata*,* C. aspera*,* A. hyacinthus*,* D. favus* and *P. sinensis*, although the specific genera involved and strength of these associations varies across coral species (Table S10). In particular, the two unassigned *Rhodobacteraceae* ASVs associated with low *C. aspera* settlement were found in at least one sample for every CCA across a range of settlement levels, but were particularly abundant in low settlement samples, especially in *L. proliferum* (MRA: 1.95 ± 0.5%) (Figures S5). However, since these unassigned *Rhodobacteraceae* ASVs were also present in high settlement samples, they may not directly inhibit settlement and may instead co-occur with other inhibitors in low-settlement communities transitioning toward a more inductive state. In a previous study, our findings suggested that *Rhodobacteraceae* are important for shifting biofilm community succession towards a more inductive state [54]. However, since our data represents one time point, we cannot determine if the presence of *Rhodobacteraceae* would alter the present microbial community overtime to create niches for microbial inducers.

## Conclusion and future directions

Specific microorganisms associated with high or low larval settlement were identified for 14 different GBR coral species. These candidate inducers and inhibitors belonged to a diverse range of microbial taxa and represent important targets for future research to distinguish the role of the algal host versus microbiome. Nonetheless, further experimental validation will be needed to confirm the causality between these identified microorganisms and settlement success. These taxa can be tested in validation experiments using isolated mono- or mixed-species biofilms or through manipulation of CCA microbiomes by enriching potential inducers or lowering inhibitor abundance. Building on these findings, we also suggest that future research focus on the functional characterisation of the CCA microbiome and the interactions with the CCA tissue-associated metabolomes to better interrogate whether common functions or biochemical pathways, rather than taxa, are the source of microbial settlement cues. While coral larvae are responding to different combinations of microbial taxa, these may represent complementary functional pathways that trigger larval settlement. The identification of microbial inducers and inhibitors for larval settlement is critically important for our understanding of coral settlement behaviour, which would in turn inform recruitment and coral populations dynamics as microbial communities shifts under anthropogenic stressors such as human-induced climate change [7, 108]. Furthermore, this study significantly adds to our knowledge and capability to guide the development of attractive substrates for coral larval settlement to optimise large-scale reef restoration.

## Supplementary Information

Below is the link to the electronic supplementary material.

Supplementary Material 1.

Supplementary Material 2.

## Data Availability

The dataset supporting the conclusions of this article are available in the NCBI Sequence Read Archive (SRA) (https:/www.ncbi.nlm.nih.gov/sra) under the BioProject accession number PRJNA1100425.
