# Supplementary material for: Insights into the role of crustose coralline algae microbiomes on coral larval settlement in the Great Barrier Reef
Source: Environ Microbiome. 2026 May 9;21:63. doi: 10.1186/s40793-026-00907-6 (PMC13162459; doi:10.1186/s40793-026-00907-6)
Supplement: Supplementary file 2 — Supplementary Material 2. [file 40793_2026_907_MOESM2_ESM.docx]

**Supplementary Information for the paper:**

**Insights into the role of crustose coralline algae microbiomes on coral larval settlement in the Great Barrier Reef**

Abigail C. Turnlund^1,2,3^, Paul A. O’Brien^1^, Laura Rix^1^, Sophie Ferguson^4^, Nicole Webster^1,4,5^, Guillermo Diaz-Pulido^6^, Muhammad Abdul Wahab^4^, Miguel Lurgi^7^, and Inka Vanwonterghem^1,8^


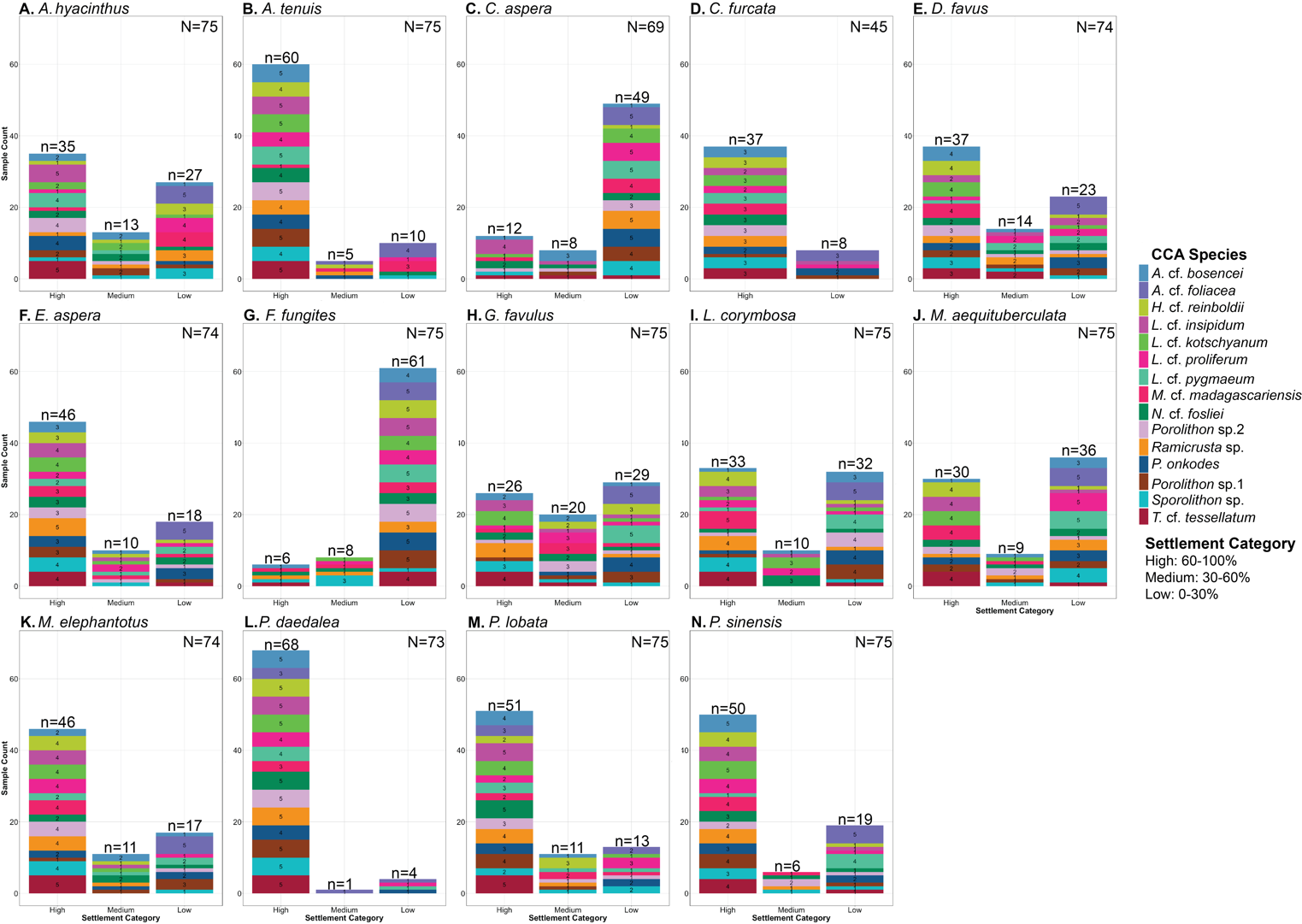


Figure S1. Distributions of crustose coralline algae (CCA) amongst settlement categories for each coral species. Each CCA sample was divided into high (60-100%), medium (30-60%), or low (0-30%) settlement categories based on its percent settlement score, with sample counts for each species shown on the y-axis.


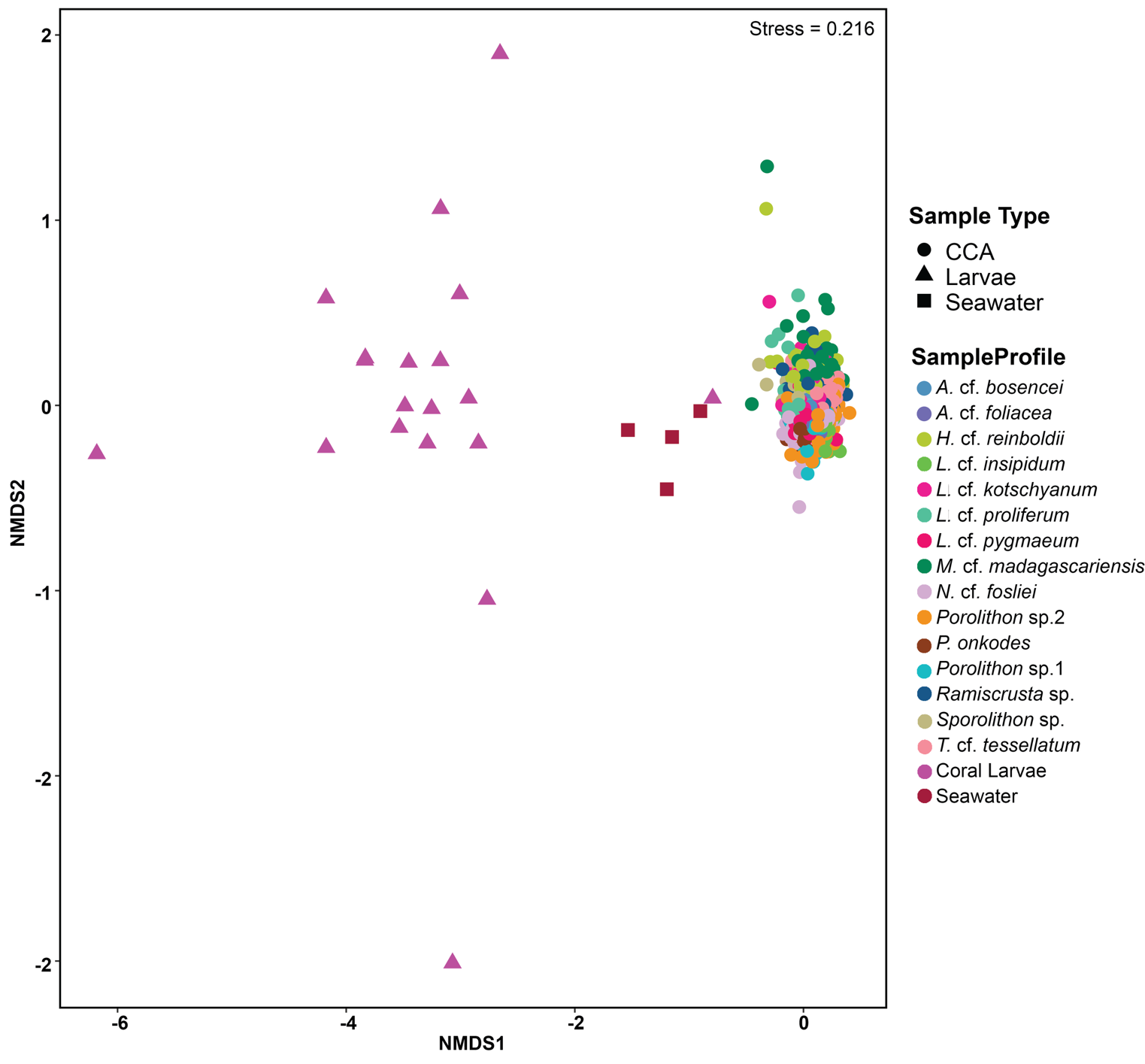


Figure S2. Microbial communities differentiate by sample type (CCA, coral larvae, and seawater). Non-metric multidimensional scaling (nMDS) ordination plots based on Bray-Curtis dissimilarity comparing microbiome composition for CCA, coral larvae, and seawater samples.

Table S1: October coral spawning species collection information adapted from M Abdul Wahab, S Ferguson, VK Snekkevik, G McCutchan, S Jeong, A Severati, CJ Randall, AP Negri and G Diaz-Pulido [1]. These corals were used in settlement assays with crustose coralline algae (CCA) collected and held in aquaria for ~1.5 weeks.

| Coral Species | Coral Family | Collection Location | Spawning Date | Spawning Time | Larval Age (days) |
| --- | --- | --- | --- | --- | --- |
| *Acropora tenuis* | Acroporidae | Palm and Magnetic Islands | 24^th^ October 2021 | 18:00 – 19:05 h | 6 |
| *Acropora anthocercis* |  | Magnetic Island | 20^th^ October 2021 | 21:16 – 23:30 h | 6 |
| *Montiopora aequituberculata* |  | Palm Islands | 25^th^ October 2021 | 19:38 – 20:50 h | 6 |
| *Echinophyllia aspera* | Lobophylliidae | Magnetic Island | 28^th^ October 2021 | 20:15 – 20:30 h | 5 |
| *Goniastrea favulus* | Merulinidae | Magnetic Island | 24^th^ October 2021 | 19:20 h | 8 |
| *Coelastrea aspera* |  |  | 24^th^ October 2021 | 21:20 – 21:34 h | 8 |
| *Dispastrea favus* |  |  | 23^rd^ October 2021 | 19:45 – 20:07 h | 4 |
| *Platygrya sinensis* |  |  | 24^th^ Octboer 2021 | 19:34 – 20:04 h | 4 |

Table S2: November coral spawning species collection information adapted from M Abdul Wahab, S Ferguson, VK Snekkevik, G McCutchan, S Jeong, A Severati, CJ Randall, AP Negri and G Diaz-Pulido [1]. These corals were used in settlement assays with crustose coralline algae (CCA) collected and held in aquaria for ~1.5 months.

| Coral Species | Coral Family | Location | Spawning Date | Spawning Time | Larval Age (days) |
| --- | --- | --- | --- | --- | --- |
| *Acropora hyacinthus* | Acroporidae | Davies Reef | 29^th^ November 2021 | 21:45 – 23:37 h | 7 |
| *Caulastrea furcata* | Merulinidae | Palm Islands | 23^rd^ November 2021 | 19:50 – 20:39 h | 6 |
| *Mycedium elephantotus* |  | Davies Reef | 24^th^ November 2021 | 20:54 h | 8 |
| *Platygyra daedalea* |  | Palm Islands | 23^rd^ November 2021 | 18:50 h | 6 |
| *Fungia fungites* | Fungiidae | National Sea Simulator aquarium facility at the Australian Institute of Marine Science captive | 25^th^ November 2021 | 18:37 – 19:26 h | 8 |
| *Lobophyllia corymbosa* | Lobophylliidae | Palm Islands | 26^th^ November 2021 | 19:27 h | 8 |
| *Porites lobata* | Poritidae | Palm Islands | 25^th^ November 2021 | 21:15 – 12:31 h | 6 |

Table S3. Crustose coralline algae (CCA) collection information adapted from M Abdul Wahab, S Ferguson, VK Snekkevik, G McCutchan, S Jeong, A Severati, CJ Randall, AP Negri and G Diaz-Pulido [1].

| CCA Species |  |  | Collection site | Sampling Depth (m) | Habitat | Light Exposure |
| --- | --- | --- | --- | --- | --- | --- |
|  | Family/Sub-family | Order |  |  |  |  |
| *Adeylithon* cf. *bosencei* | Hydrolithoideae | Corallinales | Davies Reef | 2-4 | Shallow – Deep reef | High |
| *Amphiroa* cf.  *foliacea* | Lithophylloideae | Corallinales | Davies Reef | 4 | Shallow – Mid reef | High |
| *Hydrolithon* cf. *reinboldii* | Hydrolithoideae | Corallinales | Havannah Island | 2-3 | Shallow – Deep reef | Moderate |
| *Lithophyllum* cf. *insipidium* | Lithophylloideae | Corallinales | Havannah Island | 2-3 | Crest, Shallow reef | High |
| *Lithophyllum* cf. *kotschyanum* | Lithophylloideae | Corallinales | Davies Reef | 4 | Reef Crest | High |
| *Lithothamnion* cf.  *proliferum* | Hapalidiaceae | Hapalidiales | Davies Reef | 6 | Crevices, Caves | Low |
| *Lithophyllum* cf. *pygmaeum* | Lithophylloideae | Corallinales | Davies Reef | 4 | Crest, Shallow reef | High |
| *Melyvonnea* cf. *madagascariensis* | Mesophyllumaceae | Hapalidiales | Davies Reef | 4-5 | Shallow – Deep reef | Moderate |
| *Neogoniolithon* cf. *fosliei* | Neogoniolithoideae | Corallinales | Davies Reef | 3 | Reef Crest | High |
| *Ramicrusta* sp. | Peyssonneliaceae | Peyssonneliales | Davies Reef | 4 | Crevices, Caves | Low |
| *Porolithon onkodes* | Metagoniolithoideae | Corallinales | Davies Reef | 2 | Reef Crest | High |
| *Porolithon* sp.1 | Metagoniolithoideae | Corallinales | Davies Reef | 2 | Reef Crest | High |
| *Porolithon* sp.2 | Metagoniolithoideae | Corallinales | Havannah Island | 3 | Reef Crest | High |
| *Sporolithon sp.* | Sporolithaceae | Sporolithales | Davies Reef | 6 | Crevices, Caves | Low |
| *Titanoderma* cf. *tessellatum* | Lithophylloideae | Corallinales | Davies Reef | 4 | Shallow – Deep reef | Moderate |

Table S4. Settlement assay sample settlement results (%) for each coral species per each crustose coralline algae (CCA) species. Samples chosen for 16S amplicon sequencing, removed after extraction, and removed after sequencing are denoted with an asterisk in the respective column.

*See Supplementary File 2 for full table.*

Table S5. Regression random forest model performance metrics. The ‘Mean of squared residuals’ assesses the model’s error rate and the ‘% Variation Explained’ represents the percentage of variance explained from percent settlement scores.

| Coral Species | Mean of Squared Residuals | % Variation Explained |
| --- | --- | --- |
| *A. hyacinthus* | 694.33 | 27.28 |
| *A. tenuis* | 723.57 | 8.51 |
| *C. aspera* | 803.71 | 21.34 |
| *C. furcata* | 830.61 | 16.96 |
| *D. favus* | 824.40 | 27.93 |
| *E. aspera* | 1013.51 | 16.29 |
| *F. fungites* | 436.54 | 15.65 |
| *G. favulus* | 816.24 | 21.57 |
| *L. corymbosa* | 812.59 | 18.47 |
| *M. aequituberculata* | 1106.17 | 14.93 |
| *M. elephantotus* | 805.56 | 35.26 |
| *P. daedalea* | 333.38 | 18.94 |
| *P. lobata* | 996.48 | -9.96 |
| *P. sinensis* | 839.36 | 29.56 |

Table S6. Permutational multivariate analysis of variance (PERMANOVA) statistics comparing the microbiomes of different sample types (crustose coralline algae, coral larvae, and filtered sea water).

|  | df | SS | R2 | MS | F Model | p value |
| --- | --- | --- | --- | --- | --- | --- |
| Sample Type | 2 | 5.45 | 0.01 | 2.73 | 7.16 | 0.001 |
| Residuals | 1042 | 396.87 | 0.99 | 0.38 |  |  |
| Total | 1044 | 402.33 | 1 |  |  |  |

Table S7: Permutational multivariate analysis of variance (PERMANOVA) statistics comparing the microbiome of each crustose coralline algae (CCA) sample and the settlement category it belongs to per coral species.

| *A. hyacinthus* | df | SS | R2 | MS | F Model | p value |
| --- | --- | --- | --- | --- | --- | --- |
| CCA Species | 14 | 10.21 | 0.4 | 0.73 | 2.99 | 0.001 |
| Settlement Category | 2 | 0.57 | 0.02 | 0.29 | 1.18 | 0.095 |
| CCA Species: Settlement Category | 17 | 4.63 | 0.18 | 0.27 | 1.12 | 0.004 |
| Residuals | 41 | 10 | 0.39 | 0.24 |  |  |
| Total | 74 | 25.41 | 1 |  |  |  |
|  |  |  |  |  |  |  |
| *A. tenuis* |  |  |  |  |  |  |
| CCA Species | 14 | 11.21 | 0.44 | 0.8 | 3.45 | 0.001 |
| Settlement Category | 2 | 0.48 | 0.02 | 0.24 | 1.03 | 0.348 |
| CCA Species: Settlement Category | 7 | 1.83 | 0.07 | 0.26 | 1.13 | 0.017 |
| Residuals | 51 | 11.85 | 0.47 | 0.23 |  |  |
| Total | 74 | 25.37 | 1 |  |  |  |
|  |  |  |  |  |  |  |
| *C. aspera* |  |  |  |  |  |  |
| CCA Species | 14 | 12.32 | 0.5 | 0.88 | 3.82 | 0.001 |
| Settlement Category | 2 | 0.46 | 0.02 | 0.23 | 0.99 | 0.488 |
| CCA Species: Settlement Category | 11 | 2.47 | 0.1 | 0.22 | 0.98 | 0.64 |
| Residuals | 41 | 9.44 | 0.38 | 0.23 |  |  |
| Total | 68 | 24.68 | 1 |  |  |  |
|  |  |  |  |  |  |  |
| *C. furcata* |  |  |  |  |  |  |
| CCA Species | 14 | 8.02 | 0.53 | 0.57 | 2.36 | 0.001 |
| Settlement Category | 1 | 0.23 | 0.01 | 0.23 | 0.94 | 0.613 |
| CCA Species: Settlement Category | 3 | 0.66 | 0.04 | 0.22 | 0.9 | 0.865 |
| Residuals | 26 | 6.3 | 0.41 | 0.24 |  |  |
| Total | 44 | 15.21 | 1 |  |  |  |
|  |  |  |  |  |  |  |
| *D. favus* |  |  |  |  |  |  |
| CCA Species | 14 | 11.35 | 0.43 | 0.81 | 3.28 | 0.001 |
| Settlement Category | 2 | 0.68 | 0.03 | 0.34 | 1.37 | 0.006 |
| CCA Species: Settlement Category | 19 | 4.83 | 0.18 | 0.25 | 1.03 | 0.246 |
| Residuals | 38 | 9.4 | 0.36 | 0.25 |  |  |
| Total | 73 | 26.25 | 1 |  |  |  |
|  |  |  |  |  |  |  |
| *E. aspera* |  |  |  |  |  |  |
| CCA Species | 14 | 11.81 | 0.47 | 0.84 | 3.78 | 0.001 |
| Settlement Category | 2 | 0.48 | 0.02 | 0.24 | 1.07 | 0.274 |
| CCA Species: Settlement Category | 16 | 3.76 | 0.15 | 0.23 | 1.05 | 0.158 |
| Residuals | 41 | 9.16 | 0.36 | 0.22 |  |  |
| Total | 73 | 25.2 | 1 |  |  |  |
|  |  |  |  |  |  |  |
| *F. fungites* |  |  |  |  |  |  |
| CCA Species | 14 | 11.57 | 0.45 | 0.83 | 3.46 | 0.001 |
| Settlement Category | 2 | 0.48 | 0.02 | 0.24 | 1.01 | 0.447 |
| CCA Species: Settlement Category | 10 | 2.12 | 0.08 | 0.21 | 0.89 | 0.986 |
| Residuals | 48 | 11.46 | 0.45 | 0.24 |  |  |
| Total | 74 | 25.63 | 1 |  |  |  |
|  |  |  |  |  |  |  |
| *G. favulus* |  |  |  |  |  |  |
| CCA Species | 14 | 12.79 | 0.46 | 0.91 | 3.84 | 0.001 |
| Settlement Category | 2 | 0.5 | 0.02 | 0.25 | 1.06 | 0.309 |
| CCA Species: Settlement Category | 19 | 5 | 0.18 | 0.26 | 1.11 | 0.009 |
| Residuals | 39 | 9.27 | 0.34 | 0.24 |  |  |
| Total | 74 | 27.57 | 1 |  |  |  |
|  |  |  |  |  |  |  |
| *L. corymbosa* |  |  |  |  |  |  |
| CCA Species | 14 | 11.02 | 0.42 | 0.79 | 3.39 | 0.001 |
| Settlement Category | 2 | 0.65 | 0.03 | 0.33 | 1.41 | 0.006 |
| CCA Species: Settlement Category | 16 | 4.75 | 0.18 | 0.3 | 1.28 | 0.001 |
| Residuals | 41 | 9.52 | 0.37 | 0.23 |  |  |
| Total | 73 | 25.93 | 1 |  |  |  |
|  |  |  |  |  |  |  |
| *M. aequituberculata* |  |  |  |  |  |  |
| CCA Species | 14 | 11.39 | 0.43 | 0.81 | 3.41 | 0.001 |
| Settlement Category | 2 | 0.59 | 0.02 | 0.29 | 1.23 | 0.042 |
| CCA Species: Settlement Category | 15 | 4.1 | 0.16 | 0.27 | 1.14 | 0.001 |
| Residuals | 43 | 10.26 | 0.39 | 0.24 |  |  |
| Total | 74 | 26.33 | 1 |  |  |  |
|  |  |  |  |  |  |  |
| *M. elephantotus* |  |  |  |  |  |  |
| CCA Species | 14 | 9.99 | 0.42 | 0.71 | 3.1 | 0.001 |
| Settlement Category | 2 | 0.58 | 0.02 | 0.29 | 1.25 | 0.03 |
| CCA Species: Settlement Category | 15 | 3.78 | 0.16 | 0.25 | 1.1 | 0.014 |
| Residuals | 42 | 9.66 | 0.4 | 0.23 |  |  |
| Total | 73 | 24 | 1 |  |  |  |
|  |  |  |  |  |  |  |
| *P. daedalea* |  |  |  |  |  |  |
| CCA Species | 14 | 10.51 | 0.42 | 0.75 | 2.87 | 0.001 |
| Settlement Category | 2 | 0.31 | 0.01 | 0.15 | 0.58 | 1 |
| CCA Species: Settlement Category | 3 | 0.56 | 0.02 | 0.19 | 0.72 | 1 |
| Residuals | 53 | 13.86 | 0.55 | 0.26 |  |  |
| Total | 72 | 25.24 | 1 |  |  |  |
|  |  |  |  |  |  |  |
| *P. lobata* |  |  |  |  |  |  |
| CCA Species | 14 | 10.51 | 0.4 | 0.75 | 2.81 | 0.001 |
| Settlement Category | 2 | 0.47 | 0.02 | 0.23 | 0.88 | 0.844 |
| CCA Species: Settlement Category | 14 | 3.73 | 0.14 | 0.27 | 1 | 0.487 |
| Residuals | 44 | 11.74 | 0.44 | 0.27 |  |  |
| Total | 74 | 26.46 | 1 |  |  |  |
|  |  |  |  |  |  |  |
| *P. sinensis* |  |  |  |  |  |  |
| CCA Species | 14 | 10.17 | 0.41 | 0.73 | 3 | 0.001 |
| Settlement Category | 2 | 0.57 | 0.02 | 0.29 | 1.19 | 0.039 |
| CCA Species: Settlement Category | 13 | 3.4 | 0.14 | 0.26 | 1.08 | 0.03 |
| Residuals | 45 | 10.88 | 0.43 | 0.24 |  |  |
| Total | 74 | 25.02 | 1 |  |  |  |

Table S8. Permutational multivariate analysis of variance (PERMANOVA) statistics comparing the microbiomes of CCA tested in October (2-3 weeks conditioning time) and November (4-6 weeks conditioning time).

|  | df | SS | R2 | MS | F Model | p value |
| --- | --- | --- | --- | --- | --- | --- |
| Sample Type | 2 | 5.53 | 0.01 | 2.77 | 7.30 | 0.001 |
| Residuals | 1029 | 389.76 | 0.99 | 0.38 |  |  |
| Total | 1031 | 395.29 | 1 |  |  |  |

Table S9: Pairwise permutational multivariate analysis of variance (PERMANOVA) statistics comparing the microbiome high and low settlement samples per coral species.

| High vs. Low Settlement | F Model | R2 | p value | Sig |
| --- | --- | --- | --- | --- |
| *A. hyacinthus* | 1.95 | 0.03 | 0.002 | * |
| *A. tenuis* | 1.67 | 0.02 | 0.001 | * |
| *C. aspera* | 1.65 | 0.03 | 0.008 | * |
| *C. furcata* | 1.49 | 0.03 | 0.005 | * |
| *D. favus* | 2.4 | 0.04 | 0.001 | * |
| *E. aspera* | 1.97 | 0.03 | 0.001 | * |
| *F. fungites* | 1.03 | 0.02 | 0.346 |  |
| *G. favulus* | 1.97 | 0.04 | 0.001 | * |
| *L. corymbosa* | 2.35 | 0.04 | 0.001 | * |
| *M. aequituberculata* | 2.23 | 0.03 | 0.001 | * |
| *M. elephantotus* | 2.56 | 0.04 | 0.001 | * |
| *P. daedalea* | 1.09 | 0.02 | 0.239 |  |
| *P. lobata* | 1.14 | 0.02 | 0.181 |  |
| *P. sinensis* | 2.27 | 0.03 | 0.001 | * |

Table S10: Amplicon sequence variants (ASVs) of interest found to be significantly correlated with high or low coral larval settlement in at least two of the four analyses performed in this study (Linear models, differential abundance analysis, indicator species analysis and/or random forest model). Results with negative values are associated with low coral larval settlement and positive values are associated with high coral larval settlement.

| Taxonomy | Coral Settlement | Linear Model Coefficient | Log Fold Change | Indicator Species Value | Random Forest Model Importance |
| --- | --- | --- | --- | --- | --- |
| ***Acidobacteriota*** |  |  |  |  |  |
| ***Thermoanaerobaculaceae*** |  |  |  |  |  |
| *Thermoanaerobaculaceae* *subgroup 10* 24265 | Low Settlement |  |  |  |  |
|  | *D. favus* | - | -1.28 ± 0.31 | - | 667.5 |
|  | *P. sinensis* | -0.10 ± 0.02 | -1.28 ± 0.31 | 0.656 | 6380.2 |
| ***Unassigned PAUC26f*** |  |  |  |  |  |
| Unassigned *PAUC26f* 61194 | High Settlement |  |  |  |  |
|  | *D. favus* | 0.61 ± 0.17 | 0.91± 0.33 | 0.544 | 833.4 |
|  | *G. favulus* | 0.62 ± 0.13 | - | - | 5899.4 |
|  |  |  |  |  |  |
| ***Actinobacteriota*** |  |  |  |  |  |
| ***Microtrichaceae*** |  |  |  |  |  |
| *Sva09966 marine group* 23168 | Low Settlement |  |  |  |  |
|  | *P. lobata* | - | - | 0.657 | 652.9 |
|  | *P. sinensis* | - | -1.43 ± 0.39 | 0.773 | 773.2 |
|  |  |  |  |  |  |
| ***Bacteroidota*** |  |  |  |  |  |
| ***Cyclobacteriaceae*** |  |  |  |  |  |
| *Fulvivirga* 9722 | Low Settlement |  |  |  |  |
|  | *C. furcata* | -0.70 ± 0.18 | -2.52 ± 0.45 | 0.775 | 1542.7 |
| ***Flavobacteriaceae*** |  |  |  |  |  |
| *Aquimarina* 1519 | Low Settlement |  |  |  |  |
|  | *L. corymbosa* | -0.18 ± 0.06 | - | 0.642 | 517.1 |
| *Flagellimonas* 9355 | Low Settlement |  |  |  |  |
|  | *E. aspera* | -0.70 ± 0.16 | -1.89 ± 0.45 | 0.618 | 2086.2 |
|  | *D. favus* | -0.49 ± 0.13 | -1.63 ± 0.36 | - | 1083.5 |
| *Maribacter* 12049 | Low Settlement |  |  |  |  |
|  | *M. elephantotus* | -0.22 ± 0.08 | -1.61 ± 0.49 | 0.796 | 625.3 |
| *Tenacibaculum* 23708 | Low Settlement |  |  |  |  |
|  | *D. favus* | -0.61 ± 0.22 | - | - | 639.4 |
|  | High Settlement |  |  |  |  |
|  | *A. tenuis* | - | - | 0.793 | 1096 |
| Unassigned *Flavobacteraiceae* 43816 | High Settlement |  |  |  |  |
|  | *C. aspera* | 0.40 ± 0.14 | - | 0.583 | 2049.1 |
| ***Hyphomicrobiaceae*** |  |  |  |  |  |
| *Filomicrobium* 9313 | Low Settlement |  |  |  |  |
|  | *E. aspera* | -0.15 ± 0.06 | -0.99 ± 0.28 | - | - |
| *Filomicrobium* 9329 | High Settlement |  |  |  |  |
|  | *D. favus* | 0.91 ± 0.17 | 2.10 ± 0.44 | 0.678 | 4709.3 |
|  | *E. aspera* | 0.59 ± 0.17 | 2.03 ± 0.54 | 0.641 | 667.9 |
|  | *L. corymbosa* | 0.15 ± 0.05 | - | - | 433.4 |
|  | *M. elephantotus* | 0.22 ± 0.05 | - | - | 1936.5 |
|  | *P. sinensis* | 0.17 ± 0.05 | 2.10 ± 0.44 | - | 481 |
| *Filomicrobium* 9337 | Low Settlement |  |  |  |  |
|  | *D. favus* | -0.58 ± 0.12 | -2.49 ± 0.36 | 0.655 | 4935.8 |
|  | *G. favulus* | - | - | 0.584 | 503.1 |
|  | *M. elephantotus* | -0.15 ± 0.03 | -2.12 ± 0.44 | 0.639 | 1345.9 |
|  | *E. aspera* | -0.42 ± 0.13 | -2.13 ± 0.50 | 0.711 | - |
|  | *P. sinensis* | - | -2.49 ± 0.36 | 0.592 | - |
| ***Saprospiraceae*** |  |  |  |  |  |
| *Lewinella* 11613 | Low Settlement |  |  |  |  |
|  | *M. aequituberculata* | - | - | 0.654 | 1900.6 |
| *Lewinella* 11670 | Low Settlement |  |  |  |  |
|  | *D. favus* | - | -1.34 ± 0.48 | 0.667 | - |
|  | *M. aequituberculata* | - | -0.21 ± 0.06 | 0.667 | 3494.9 |
|  |  |  |  |  |  |
| ***Bdellovibrionota*** |  |  |  |  |  |
| ***Bdellovibrionaceae*** |  |  |  |  |  |
| *Bdellovibrio* 2487 | High Settlement |  |  |  |  |
|  | *M. elephantotus* | 0.24 ± 0.06 | 1.10 ± 0.34 | - | 1092.7 |
|  |  |  |  |  |  |
| ***Crenarchaeota*** |  |  |  |  |  |
| ***Nitrosopumilaceae*** |  |  |  |  |  |
| Candidatus *Nitrosopumilus* 5680 | High Settlement |  |  |  |  |
|  | *D. favus* | 1.01 ± 0.24 | 1.43 ± 0.48 | - | 499.7 |
|  | *P. sinensis* | 0.22 ± 0.09 | 1.43 ± 0.48 | - | - |
| Candidatus *Nitrosopumilus* 5799 | High Settlement |  |  |  |  |
|  | *D. favus* | 1.12 ± 0.37 | - | 0.573 | - |
|  | *L. corymbosa* | 0.11 ± 0.04 | - | - | 551.2 |
|  | *P. sinensis* | 0.35 ± 0.07 | - | 0.694 | 2306.6 |
|  |  |  |  |  |  |
| ***Desulfobacterota*** |  |  |  |  |  |
| Unassigned *PB19* 61608 | Low Settlement |  |  |  |  |
|  | *D. favus* | -0.600 ± 0.17 | -1.60 ± 0.37 | 0.708 | 648.8 |
|  | *P. sinensis* | - | -1.60 ± 0.37 | 0.633 | - |
|  |  |  |  |  |  |
| ***Planctomycetota*** |  |  |  |  |  |
| ***Phycisphaeraceae*** |  |  |  |  |  |
| *Urania-1B-19 marine sediment group* 79403 | Low Settlement |  |  |  |  |
|  | *C. furcata* | -0.35 ± 0.07 | -1.39 ± 0.34 | 0.697 | 1619 |
| *Urania-1B-19 marine sediment group* 79717 | High Settlement |  |  |  |  |
|  | *F. fungites* | 0.39 ± 0.14 | - | 0.619 | - |
| *Blastopirellula* 3098 | Low Settlement |  |  |  |  |
|  | *M. elephantotus* | -0.18 ± 0.04 | -1.84 ± 0.35 | 0.746 | 30118.2 |
| *Pir4 lineage* 17037 | Low Settlement |  |  |  |  |
|  | *D. favus* | - | -1.20 ± 0.37 | - | 503.3 |
| *Pir4 lineage* 17140 | Low Settlement |  |  |  |  |
|  | *M. elephantotus* | -0.21 ± 0.06 | -2.54 ± 0.55 | 0.758 | 4383.2 |
| ***Rubinisphaeraceae*** |  |  |  |  |  |
| Unassigned *Rubinisphaeraceae* 68411 | Low Settlement |  |  |  |  |
|  | *D. favus* | -0.30 ± 0.13 | -1.76 ± 0.41 | 0.663 | 1204.9 |
| **Unassigned *OM190*** |  |  |  |  |  |
| Unassigned *OM190* 59838 | Low Settlement |  |  |  |  |
|  | *E. aspera* | -0.43 ± 0.15 | -1.16 ± 0.34 | 0.697 | 510.7 |
| Unassigned *OM190* 57962 | Low Settlement |  |  |  |  |
|  | *A. hyacinthus* | -0.65 ± 0.17 | - | 0.591 | 614.7 |
|  | *M. elephantotus* | - | -1.35 ± 0.37 | 0.759 | - |
|  |  |  |  |  |  |
| ***Proteobacteria*** |  |  |  |  |  |
| ***Granulosicoccaceae*** |  |  |  |  |  |
| *Granulosicoccus* 9935 | Low Settlement |  |  |  |  |
|  | *C. furcata* | -0.58 ± 0.10 | -1.78 ± 0.45 | 0.755 | 2082.7 |
|  | *M. elephantotus* | - | -1.64 ± 0.4 | 0.757 | 2953.1 |
|  | High Settlement |  |  |  |  |
|  | *P. daedalea* | 0.35 ± 0.11 | - | - | 334.9 |
| ***Kiloniellaceae*** |  |  |  |  |  |
| *Limibacillus* 11732 | High Settlement |  |  |  |  |
|  | *D. favus* | 0.53 ± 0.17 | 1.27 ± 0.38 | 0.583 | 824.1 |
|  | *P. sinensis* | 0.21 ± 0.06 | 1.27 ± 0.38 | - | - |
| *Limibaculum* 11762 | Low Settlement |  |  |  |  |
|  | *D. favus* | - | -1.65 ± 0.45 | 0.692 | - |
|  | *L. corymbosa* | -0.19 ± 0.06 | - | 0.704 | 1037.6 |
|  | *P. sinensis* | - | -1.65 ± 0.45 | 0.666 | - |
| *Pelagibius* 15321 | Low Settlement |  |  |  |  |
|  | *A. tenuis* | - | -1.72 ± 0.39 | 0.631 | 1240.4 |
| ***Marinobacteraceae*** |  |  |  |  |  |
| *Marinobacter* 12244 | High Settlement |  |  |  |  |
|  | *G. favulus* | - | - | 0.587 | 496.3 |
|  | *M. elephantotus* | 0.20 ± 0.05 | 1.59 ± 0.42 | 0.615 | 783.5 |
| ***Methyloligellaceae*** |  |  |  |  |  |
| Unassigned *Methyloligellaceae* 52562 | High Settlement |  |  |  |  |
|  | *C. aspera* | 0.58 ± 0.13 | - | 0.594 | 1031.4 |
| ***Nitrincolaceae*** |  |  |  |  |  |
| *Neptuniibacter* 13322 | High Settlement |  |  |  |  |
|  | *G. favulus* | 0.44 ± 0.12 | - | - | 651.1 |
|  | *M. elephantotus* | 0.26 ± 0.08 | 1.98 ± 0.63 | - | - |
|  | *P. sinensis* | 0.10 ± 0.03 | - | - | 389.8 |
| *Neptuniibacter* 13326 | High Settlement |  |  |  |  |
|  | *L. corymbosa* | 0.18 ± 0.07 | - | - | 3894.6 |
|  | *M. elephantotus* | 0.17 ± 0.07 | - | 0.665 | 1640.6 |
| *Neptuniibacter* 13337 | High Settlement |  |  |  |  |
|  | *P. sinensis* | 0.19 ± 0.07 | - | 0.809 | 1005.7 |
| ***Rhizobiaceae*** |  |  |  |  |  |
| *Lentilitoribacter* 11308 | Low Settlement |  |  |  |  |
|  | *C. aspera* | - | - | 0.737 | 465.4 |
|  | *D. favus* | - | -1.30 ± 0.39 | - | 1177.6 |
|  | *L. corymbosa* | - | - | 0.654 | 467.6 |
| Unassigned *Rhizobiaceae* 66456 | Low Settlement |  |  |  |  |
|  | *D. favus* | -0.43 ± 0.17 | -1.84 ± 0.5 | 0.55 | 475.4 |
|  | *P. sinensis* | - | -1.84 ± 0.5 | 0.619 | - |
| Unassigned *Rhizobiaceae* 66482 | High Settlement |  |  |  |  |
|  | *G. favulus* | 0.68 ± 0.2 | - | 0.668 | 2035.1 |
| ***Rhodobacteraceae*** |  |  |  |  |  |
| *Roseovarius* 20547 | Low Settlement |  |  |  |  |
|  | *C. furcata* | -0.61 ± 0.17 | -2.06 ± 0.54 | 0.808 | 535.3 |
| *Ruegeria* 20979 | Low Settlement |  |  |  |  |
|  | *M. aequituberculata* | -0.10 ± 0.03 | -1.82 ± 0.41 | - | 4312.8 |
| Unassigned *Rhodobacteraceae* 66734 | Low Settlement |  |  |  |  |
|  | *C. aspera* | - | - | 0.715 | 709.2 |
| Unassigned *Rhodobacteraceae* 67059 | Low Settlement |  |  |  |  |
|  | *A. hyacinthus* | -0.57 ± 0.15 | -1.93 ± 0.45 | 0.667 | 4137 |
|  | *D. favus* | -0.37 ± 0.13 | -1.37 ± 0.49 | - | - |
|  | *P. sinensis* | -0.09 ± 0.04 | -1.37 ± 0.49 | - | - |
| Unassigned *Rhodobacteraceae* 67244 | Low Settlement |  |  |  |  |
|  | *C. aspera* | -0.67 ± 0.20 | - | 0.812 | 2988.9 |
|  | *D. favus* | -0.43 ± 0.17 | -1.71 ± 0.55 | - | 797.4 |
| **Unassigned *Alphaproteobacteria*** |  |  |  |  |  |
| Unassigned *Alphaproteobacteria* 27920 | High Settlement |  |  |  |  |
|  | *E. aspera* | 0.79 ± 0.20 | - | 0.63 | - |
| **Unassigned *Gammaproteobacteria*** |  |  |  |  |  |
| Unassigned *Gammaproteobacteria* 44435 | Low Settlement |  |  |  |  |
|  | *C. furcata* | -1.06 ± 0.27 | -2.41 ± 0.60 | 0.866 | 878.6 |
|  | *G. favulus* | -0.37 ± 0.12 | - | - | 881.2 |
| Unassigned *Gammaproteobacteria* 45636 | Low Settlement |  |  |  |  |
|  | *C. furcata* | -0.56 ± 0.09 | -1.86 ± 0.42 | 0.764 | 2113.6 |
|  | *M. elephantotus* | -0.13 ± 0.04 | -1.50 ± 0.34 | 0.672 | 2006 |
| **Unassigned *KI89A*** |  |  |  |  |  |
| Unassigned *KI89A* 50048 | Low Settlement |  |  |  |  |
|  | *A. tenuis* | - | -1.70 ± 0.45 | 0.603 | 720.2 |
| **Unassigned *EC3*** |  |  |  |  |  |
| Unassigned *EC3* 42202 | Low Settlement |  |  |  |  |
|  | *D. favus* | -0.31 ± 0.13 | -1.25 ± 0.38 | - | - |
|  | *G. favulus* | -0.57 ± 0.19 | - | 0.558 | 1366.8 |
|  | *P. sinensis* | -0.20 ± 0.05 | -1.25 ± 0.38 | 0.759 | 4008.1 |
| **Unassigned *Thalassobaculales*** |  |  |  |  |  |
| Unassigned *Thalassobaculales* 75494 | High Settlement |  |  |  |  |
|  | *A. hyacinthus* | - | 1.73 ± 0.42 | 0.62 | 3725.7 |
|  |  |  |  |  |  |
| **Unassigned *Bacteria*** |  |  |  |  |  |
| Unassigned *Bacteria* 33069 | Low Settlement |  |  |  |  |
|  | *E. aspera* | -0.43 ± 0.13 | -0.99 ± 0.28 | 0.682 | 1833.9 |
|  | *P. sinensis* | -0.10 ± 0.04 | - | 0.641 | - |
|  |  |  |  |  |  |
| ***Verrucomicrobiota*** |  |  |  |  |  |
| ***Rubritaleaceae*** |  |  |  |  |  |
| *Roseibacillus* 20376 | Low Settlement |  |  |  |  |
|  | *C. furcata* | -0.77 ± 0.24 | -3.10 ± 0.72 | 0.903 | 1384.6 |
|  | *P. sinensis* | -0.29 ± 0.06 | - | 0.722 | 5613.9 |
| *Rubritalea* 20963 | Low Settlement |  |  |  |  |
|  | *G. favulus* | -0.85 ± 0.26 | - | - | 1185.3 |

***Individual Coral Species Results***

***Acropora hyacinthus***

There was a significant difference between the high and low settlement crustose coralline algae (CCA) microbial communities (PERMANOVA: F=1.95, p<0.011), with specific amplicon sequence variants (ASVs) found from a combination of linear models, differential abundance, indicator species, and random forest (RF) analyses. Two ASVs were associated with high settlement and are found across most high settlement samples, regardless of CCA species (Figure S3). Six ASVs were associated with low settlement are found in specific low settlement CCA species (Figure S3). While most ASVs associated with low settlement were identified as low settlement indicators and had varying levels of RT model importance values, an unassigned *Rhodobacteraceae* ASV was identified in all four methods and is most abundant in *A.* cf. *foliacea* samples (Figure S3).


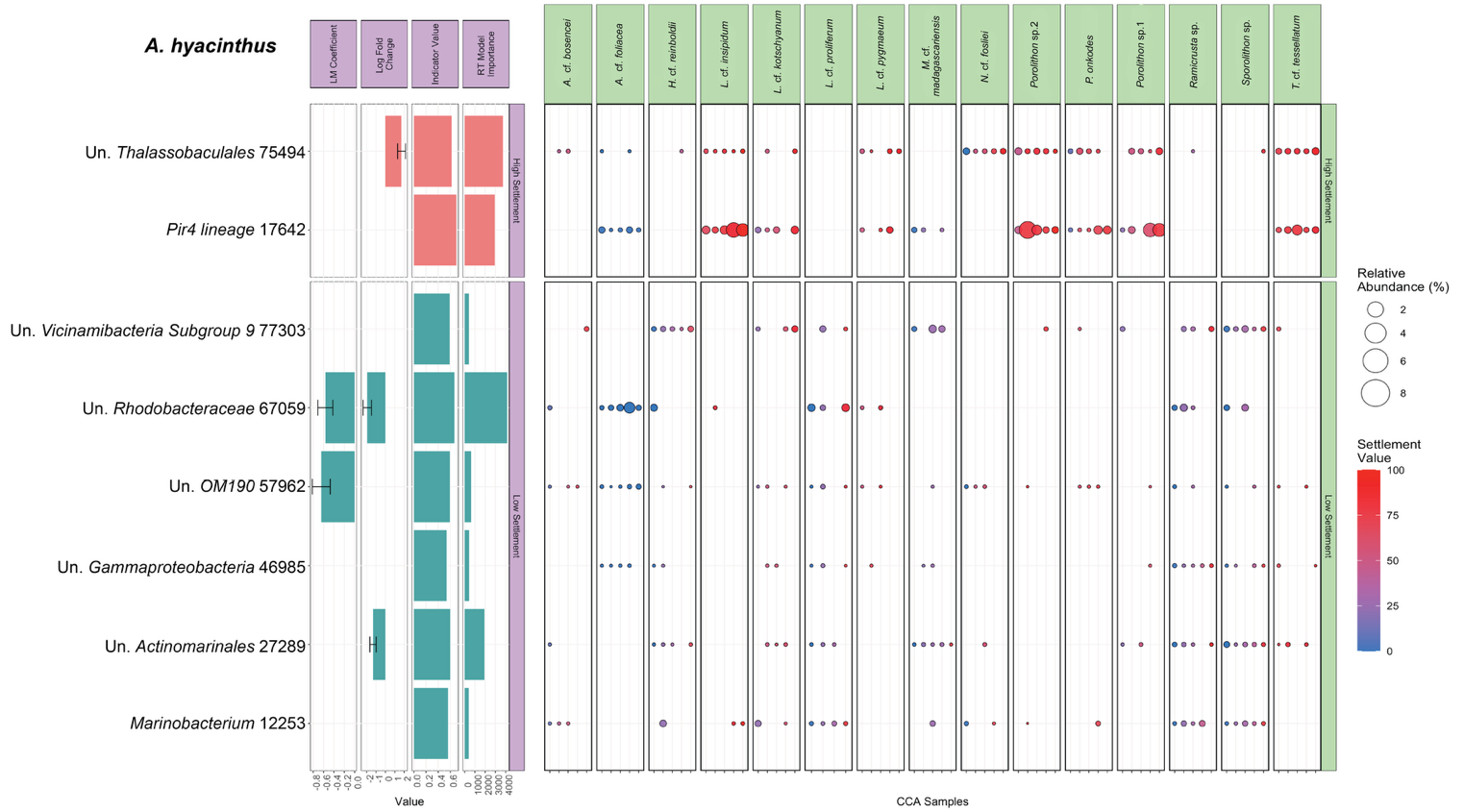


Figure S3. Amplicon sequence variants (ASVs) of interest associated with high and low *A. hyacinthus* settlement. ASVs were deemed of interest if they had significant results in two out of the four methods used (Linear models, differential abundance, indicator species, and random forest analyses). Results are visualised as bars for each ASV, and the greater the value the stronger the association between the ASV and settlement. ASV distribution is visualised across all samples grouped by crustose coralline algae in the bubble plot. The size of the bubble corresponds to the ASVs relative abundance, and the colour refers to the settlement score in the given sample (Red = high settlement, Blue = low settlement). Each ASV is labeled with a unique number to differentiate individual ASVs across the entire dataset.

***Acropora tenuis***

There was a significant difference between the high and low settlement crustose coralline algae (CCA) microbial communities (PERMANOVA: F=1.67, p<0.006), with specific amplicon sequence variants (ASVs) found from a combination of linear models, differential abundance, indicator species, and random forest (RF) analyses. Four ASVs were associated with low settlement were mainly present in *A.* cf. *foliacea* low settlement samples (Figure S4). These low settlement taxa consisted of a diverse group of taxa with an unassigned *KI89A* clade ASV having the highest relative abundance across samples and found to be differentially abundant, an indicator, and predicted to be associated/of low settlement.


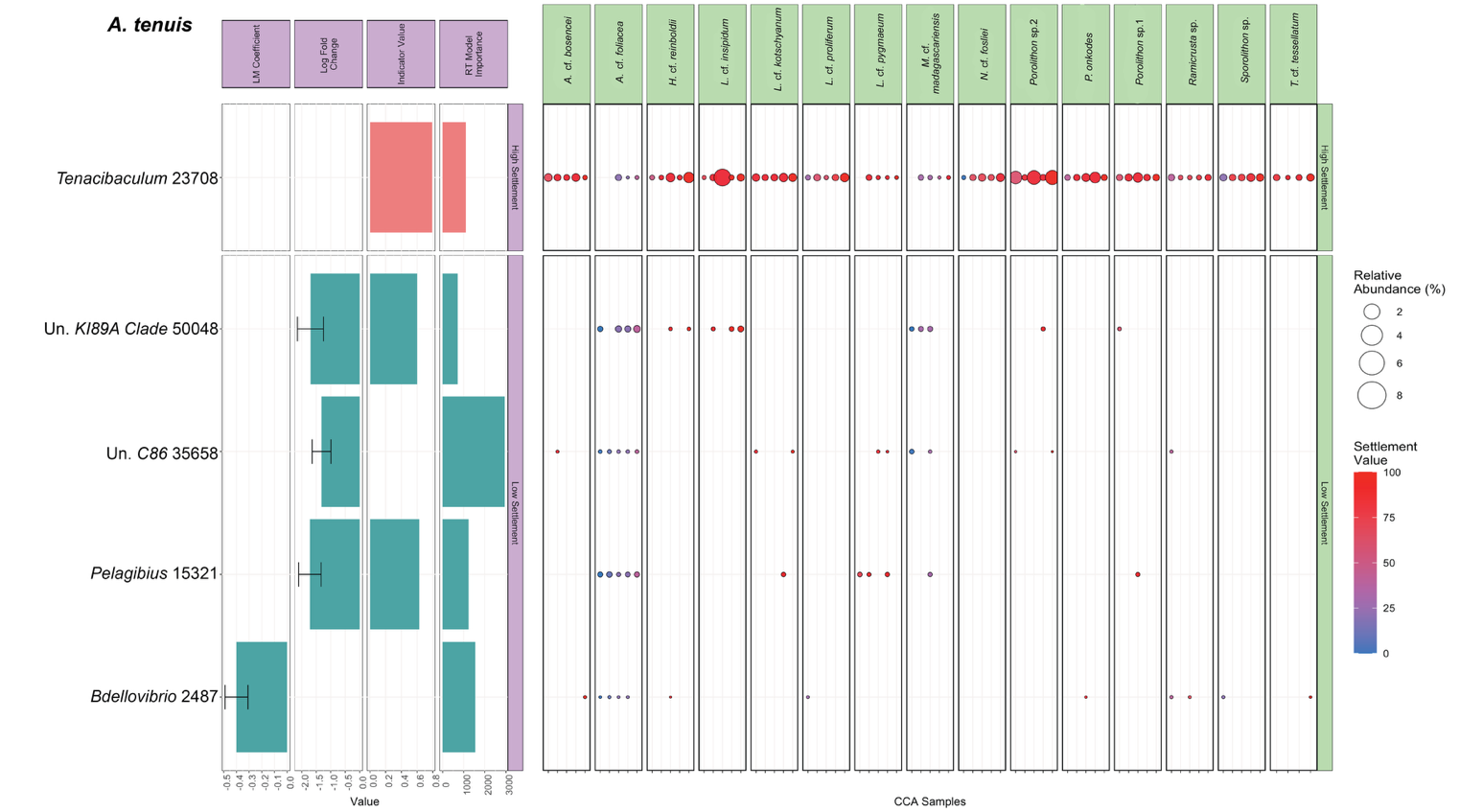


Figure S4. Amplicon sequence variants (ASVs) of interest associated with high and low *A. tenuis* settlement. ASVs were deemed of interest if they had significant results in two out of the four methods used (Linear models, differential abundance, indicator species, and random forest analyses). Method results are visualised as bars for each ASV, and the greater the method value the more associated the ASV is with high or low settlement samples. ASV distribution is visualised across all samples grouped by crustose coralline algae in the bubble plot. The size of the bubble corresponds to the ASVs relative abundance, and the colour refers to the settlement score in the given sample (Red = high settlement, Blue = low settlement). Each ASV is labeled with an arbitrary number to differentiate individual ASVs across the entire dataset.

***Coelastrea aspera***

There was a significant difference between the high and low settlement crustose coralline algae (CCA) microbial communities (PERMANOVA: F=1.65, p<0.04), with specific amplicon sequence variants (ASVs) found from a combination of linear models, differential abundance, indicator species, and random forest (RF) analyses. Six ASVs were associated with high settlement, however, they are found sporadically across CCA samples in low abundance (Figure S5). No ASVs were found to be significantly differentially abundant between high and low settlement samples, therefore only linear model, indicator species, and random forest analyses were analysed.


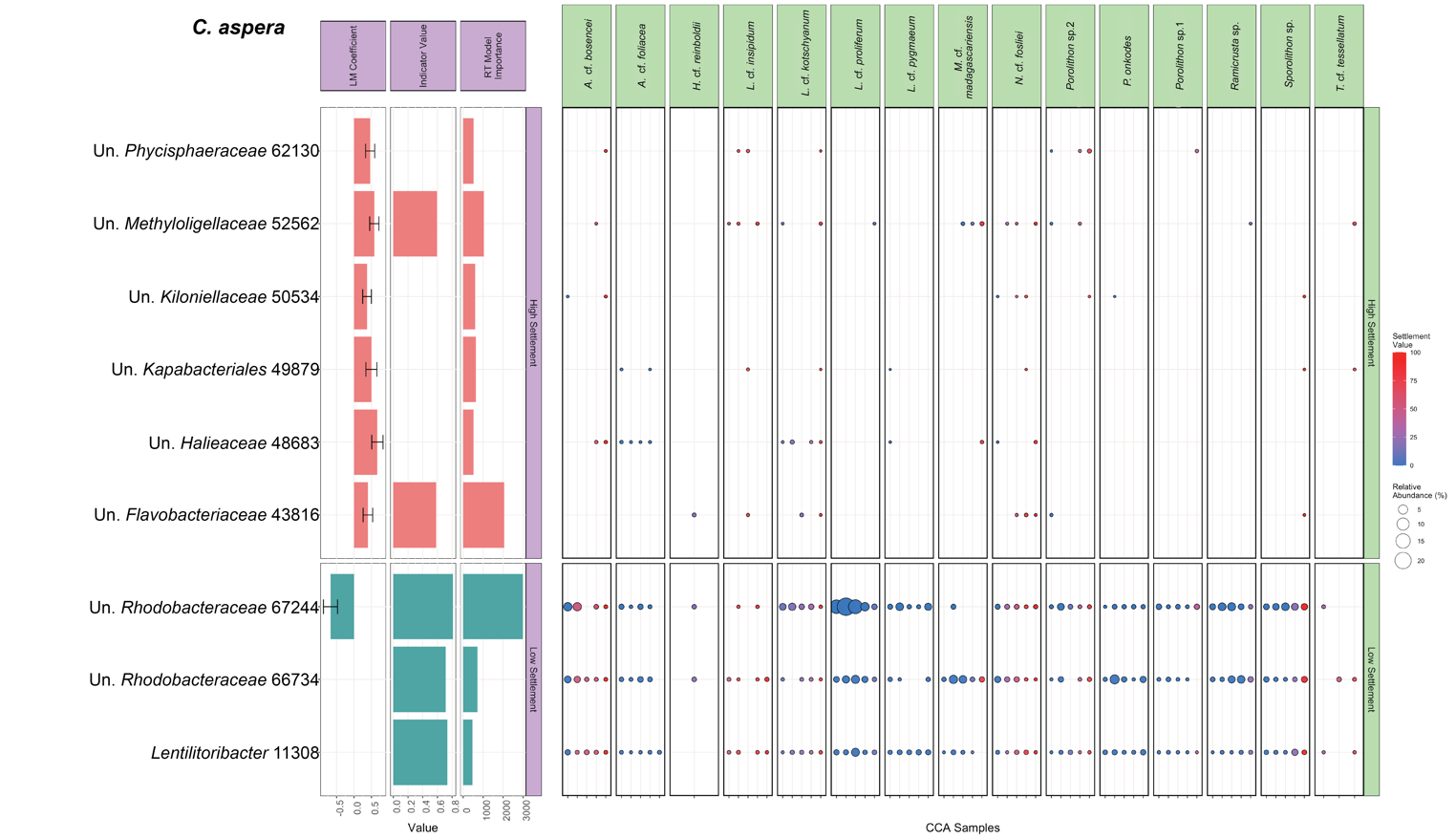


Figure S5. Amplicon sequence variants (ASVs) of interest associated with high and low *C. aspera* settlement. ASVs were deemed of interest if they had significant results in two out of the four methods used (Linear models, differential abundance, indicator species, and random forest analyses). Method results are visualised as bars for each ASV, and the greater the method value the more associated the ASV is with high or low settlement samples. No ASVs were significantly differential abundant in either high or low settlement. ASV distribution is visualised across all samples grouped by crustose coralline algae in the bubble plot. The size of the bubble corresponds to the ASVs relative abundance, and the colour refers to the settlement score in the given sample (Red = high settlement, Blue = low settlement). Each ASV is labeled with an arbitrary number to differentiate individual ASVs across the entire dataset.

***Caulastrea furcata***

There was a significant difference between the high and low settlement crustose coralline algae (CCA) microbial communities (PERMANOVA: F=1.49, p<0.005), with specific amplicon sequence variants (ASVs) found from a combination of linear models, differential abundance, indicator species, and random forest (RF) analyses. No ASVs were associated with high settlement, but 29 ASVs were associated with low settlement (Figure S6). These ASVs were largely absent from high settlement CCA such as *M.* cf. *madagascariensis, N.* cf. *fosliei, Porolithon* sp.2, and *Sporolithon* sp. (Figure S6). *Amphiroa* cf. *foliacea* and *Porolithon* sp.1 samples contained a majority of low settlement associated ASVs, with higher abundances in low settlement samples. Of these ASVs, 8 ASVs were found to be significantly associated with low settlement in all methods tested, which were associated with *Phycisphaeraceae*, *Gammaproteobacteria, Rubritaleaceae, Pirellulaceae, Granulosicoccaceae,* and *Cyclobacteriaceae* (Figures S6).


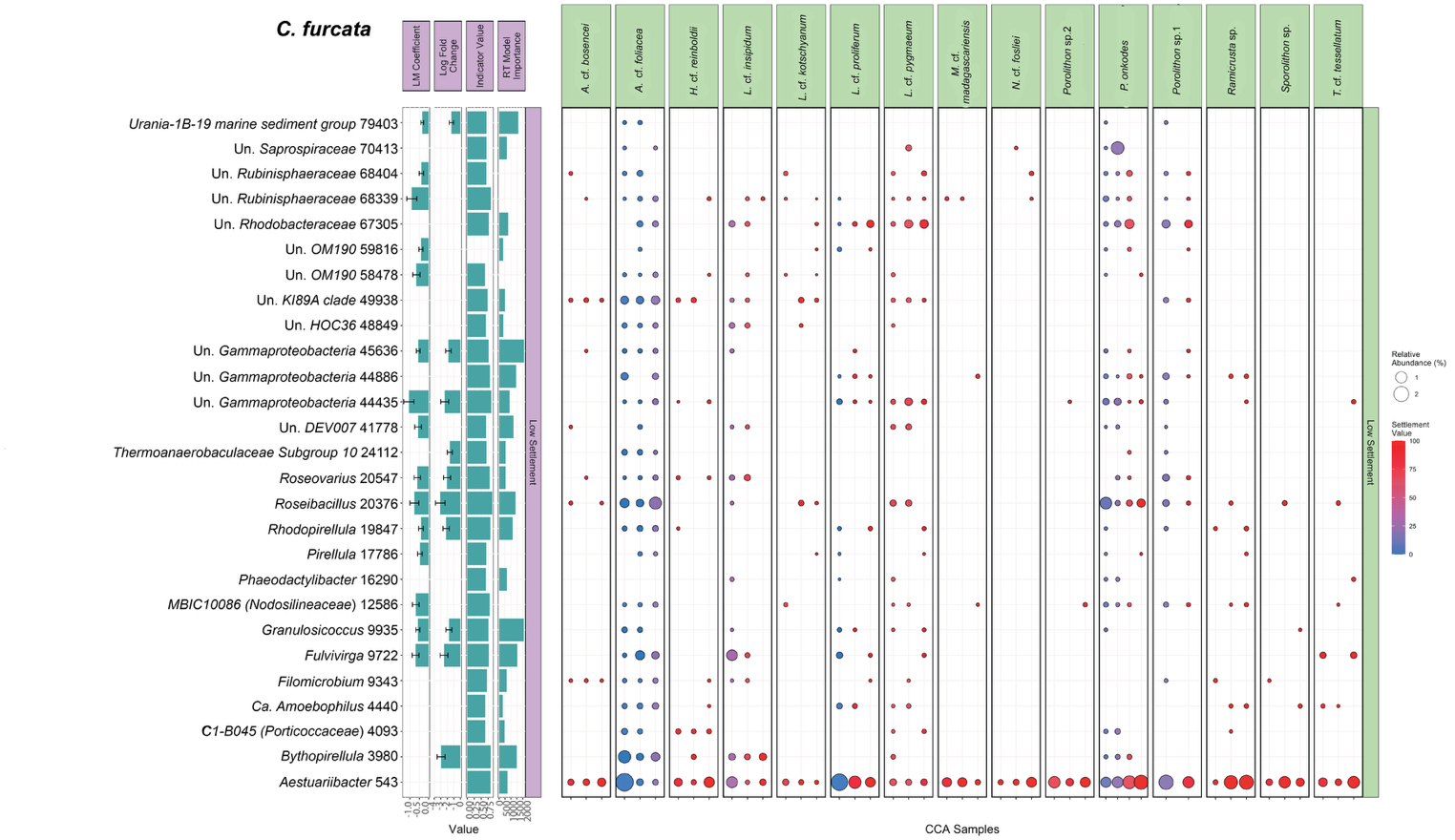


Figure S6. Amplicon sequence variants (ASVs) of interest associated with high and low *C. furcata* settlement. ASVs were deemed of interest if they had significant results in two out of the four methods used (Linear models, differential abundance, indicator species, and random forest analyses). Method results are visualised as bars for each ASV, and the greater the method value the more associated the ASV is with high or low settlement samples. ASV distribution is visualised across all samples grouped by crustose coralline algae in the bubble plot. The size of the bubble corresponds to the ASVs relative abundance, and the colour refers to the settlement score in the given sample (Red = high settlement, Blue = low settlement). Each ASV is labeled with an arbitrary number to differentiate individual ASVs across the entire dataset.

***Dipsastrea favus***

There was a significant difference between the high and low settlement crustose coralline algae (CCA) microbial communities (PERMANOVA: F=2.40, p<0.006), with specific amplicon sequence variants (ASVs) found from a combination of linear models, differential abundance, indicator species, and random forest (RF) analyses. Fourteen ASVs were associated with high settlement, which consisted of *Pirellulaceae*, *Nitrosopumilaceae, Hyphomicrobiaceae, Kiloniellaceae, Nitrospiraceae, PS1 clade* and unassigned taxa within *PAUC26f* and *Alphaproteobacteria* (Figure S7). These taxa were absent from low settlement CCA *A.* cf. *foliacea* and found in the high settlement samples of all other species of CCA (Figure S7). Sixty-one ASVs were associated with low settlement with most taxa belonging to *Planctomycetes* or *Flavobacteriaceae* (Figure S7). These taxa were largely present in *A.* cf. *foliacea* and in the low settlement samples of other CCA species, except *M.* cf. *madagascariensis* (Figure S7).


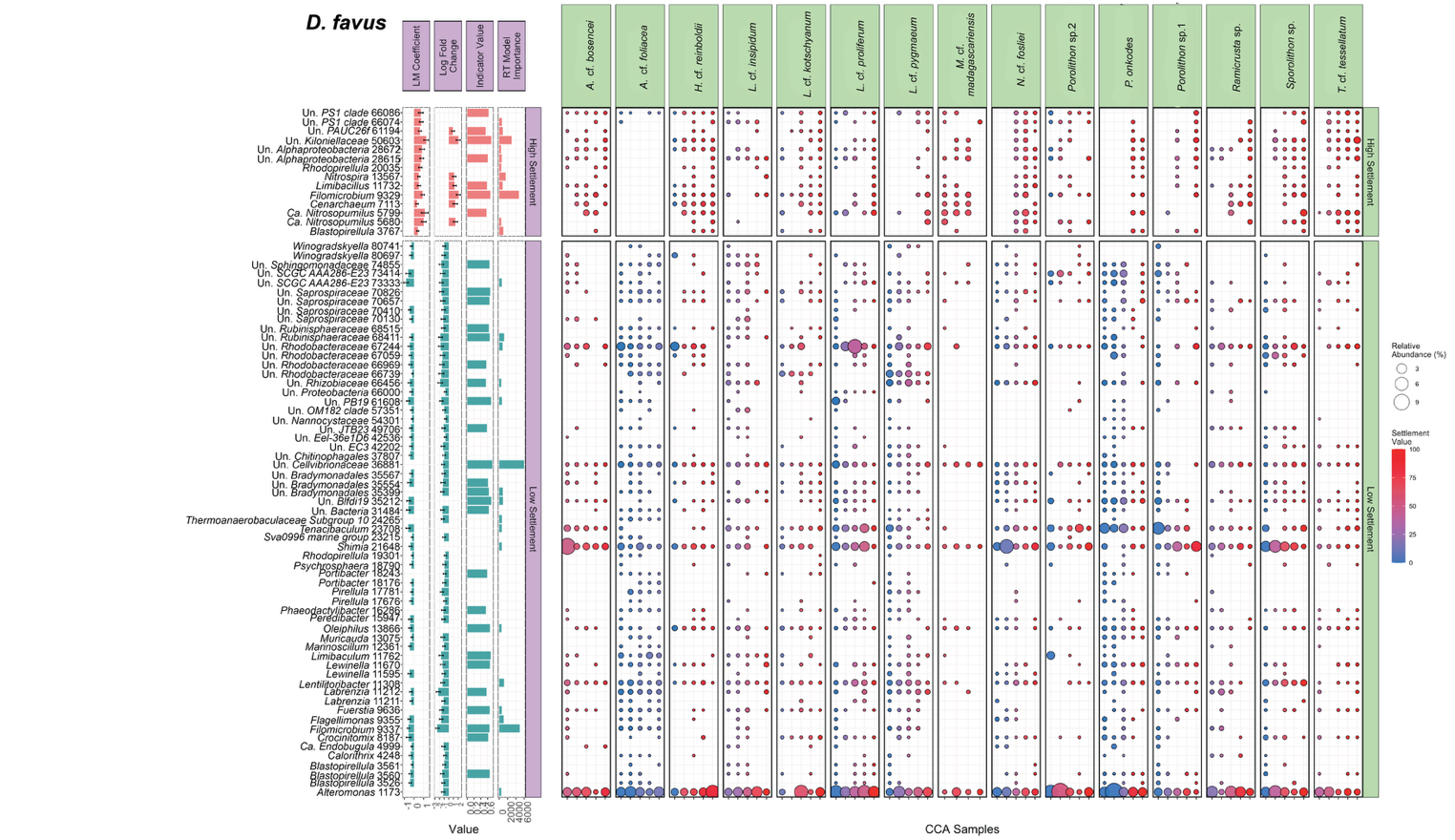


Figure S7. Amplicon sequence variants (ASVs) of interest associated with high and low *D. favus* settlement. ASVs were deemed of interest if they had significant results in two out of the four methods used (Linear models, differential abundance, indicator species, and random forest analyses). Method results are visualised as bars for each ASV, and the greater the method value the more associated the ASV is with high or low settlement samples. ASV distribution is visualised across all samples grouped by crustose coralline algae in the bubble plot. The size of the bubble corresponds to the ASVs relative abundance, and the colour refers to the settlement score in the given sample (Red = high settlement, Blue = low settlement). Each ASV is labeled with an arbitrary number to differentiate individual ASVs across the entire dataset.

***Echinophyllia aspera***

There was a significant difference between the high and low settlement crustose coralline algae (CCA) microbial communities (PERMANOVA: F=1.97, p<0.006), with specific amplicon sequence variants (ASVs) found from a combination of linear models, differential abundance, indicator species, and random forest (RF) analyses. Only two ASVs were associated with high settlement, and one ASV found to be significant for all four methods, a *Filomicrobium* ASV (Figure S8). The *Filomicrobium* ASV was found in at least one high settlement sample for every single CCA species except *A.* cf. *foliacea* and *L.* cf. *pygmaeum* (Figure S8). There were 32 ASVs associated with low settlement and consisted of a diversity of taxa including two *Filomicrobium, Pir4 lineage* (*Pirellulaceae*), and *Rubritalea* ASVs (Figure S8). *Amphiroa* cf. *foliacea* and low settlement *Porolithon* sp.2 samples had the highest number of ASVs associated with low settlement present compared to the other CCA species (Figure S8).


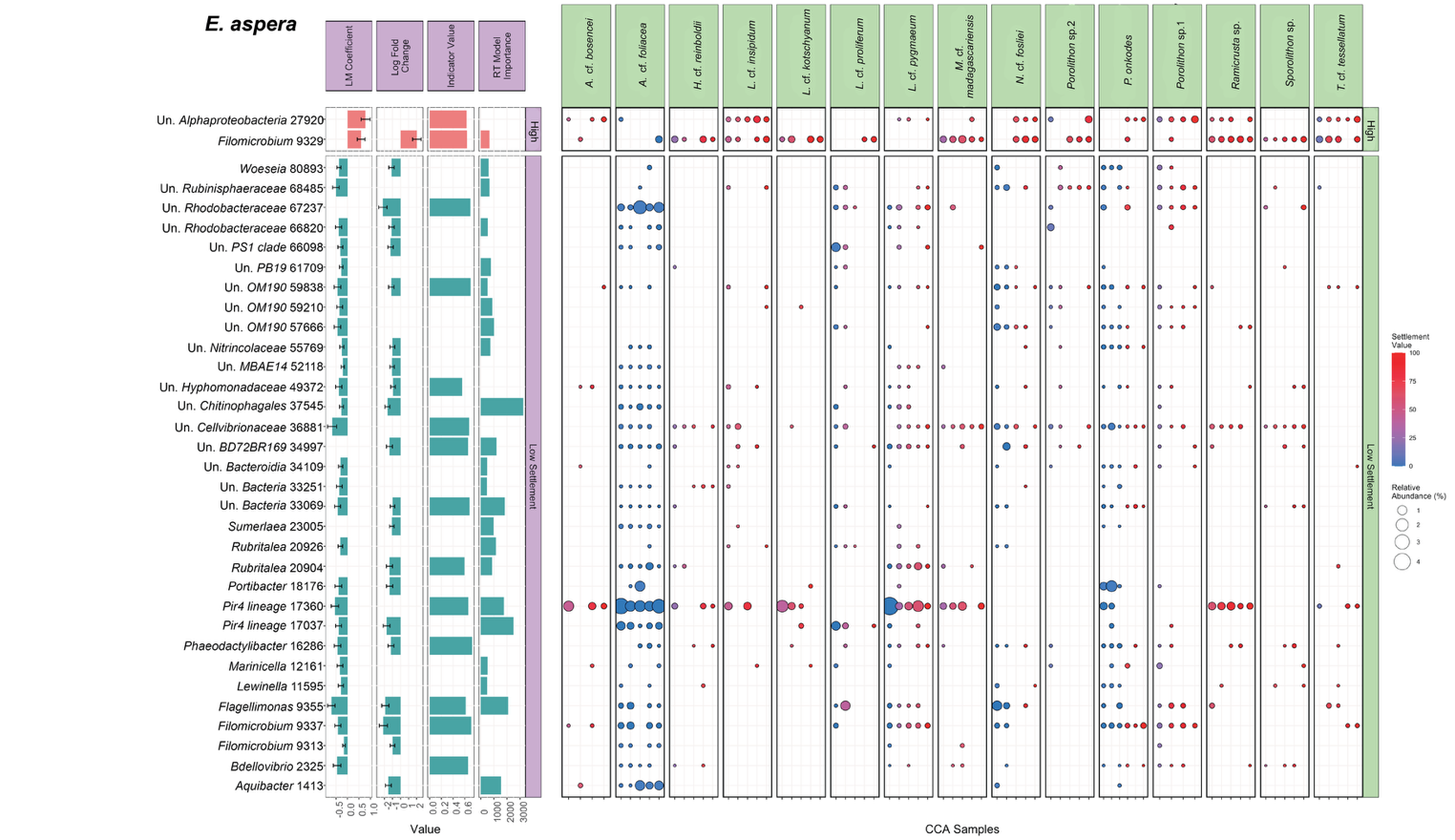


Figure S8. Amplicon sequence variants (ASVs) of interest associated with high and low *E. aspera* settlement. ASVs were deemed of interest if they had significant results in two out of the four methods used (Linear models, differential abundance, indicator species, and random forest analyses). Method results are visualised as bars for each ASV, and the greater the method value the more associated the ASV is with high or low settlement samples. ASV distribution is visualised across all samples grouped by crustose coralline algae in the bubble plot. The size of the bubble corresponds to the ASVs relative abundance, and the colour refers to the settlement score in the given sample (Red = high settlement, Blue = low settlement). Each ASV is labeled with an arbitrary number to differentiate individual ASVs across the entire dataset.

***Fungia fungites***

There was no significant difference between the high and low settlement crustose coralline algae (CCA) microbial communities (PERMANOVA: F=1.03, p=0.456) and likely not responding to microbial cues. Fifteen ASVs associated with high settlement were identified with linear models, indicator species analysis and random forest analysis, however, the sample distribution between high settlement (n = 6) and low settlement samples (n = 61) is disproportionate, and therefore might introduce some bias to these results (Figure S9).


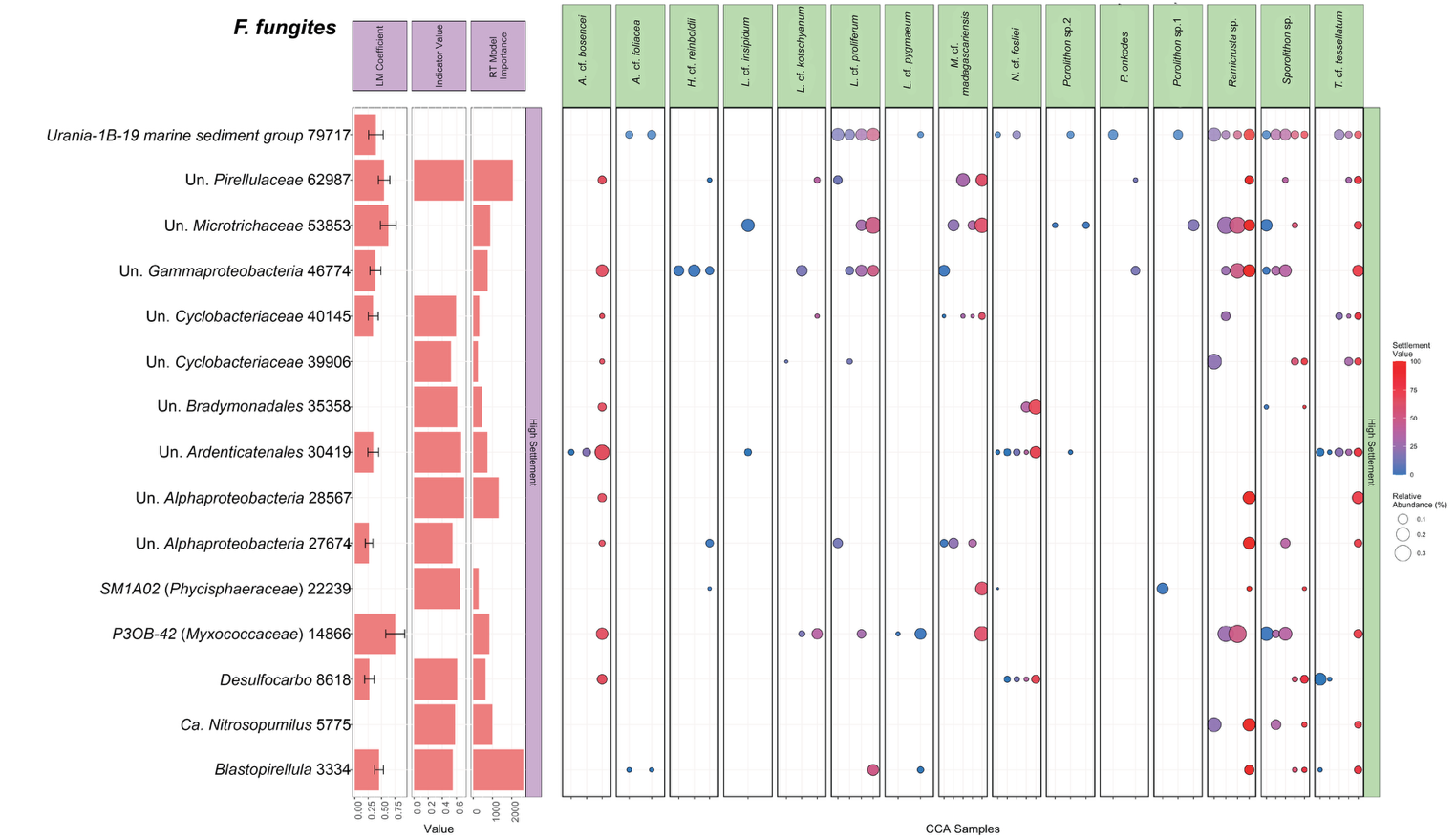


Figure S9. Amplicon sequence variants (ASVs) of interest associated with high *F. fungites* settlement. ASVs were deemed of interest if they had significant results in two out of the four methods used (Linear models, differential abundance, indicator species, and random forest analyses). Method results are visualised as bars for each ASV, and the greater the method value the more associated the ASV is with high or low settlement samples. ASV distribution is visualised across all samples grouped by crustose coralline algae in the bubble plot. The size of the bubble corresponds to the ASVs relative abundance, and the colour refers to the settlement score in the given sample (Red = high settlement, Blue = low settlement). Each ASV is labeled with an arbitrary number to differentiate individual ASVs across the entire dataset. No ASVs were identified with significant differential abundance between settlement categories.

***Goniastrea favulus***

There was a significant difference between the high and low settlement crustose coralline algae (CCA) microbial communities (PERMANOVA: F=1.97, p<0.04), with specific amplicon sequence variants (ASVs) found from a combination of linear models, differential abundance, indicator species, and random forest (RF) analyses. Six ASVs were associated with high settlement and only two of those ASVs, an unassigned *Rhizobiaceae* and *Neptuniibacter* ASV, were found in at least one sample from every single CCA species with a higher abundance in high settlement samples (Figure S10). Seven ASVs were associated with low settlement with three unassigned *Rhodobacteraceae* ASVs found in at least one sample from most CCA species (Figure S10). The other low settlement associated ASVs were more sporadic amongst the different CCA samples with them being all present in *A.* cf. *foliacea* and *L.* cf. *pygmaeum* low settlement samples (Figures S10).


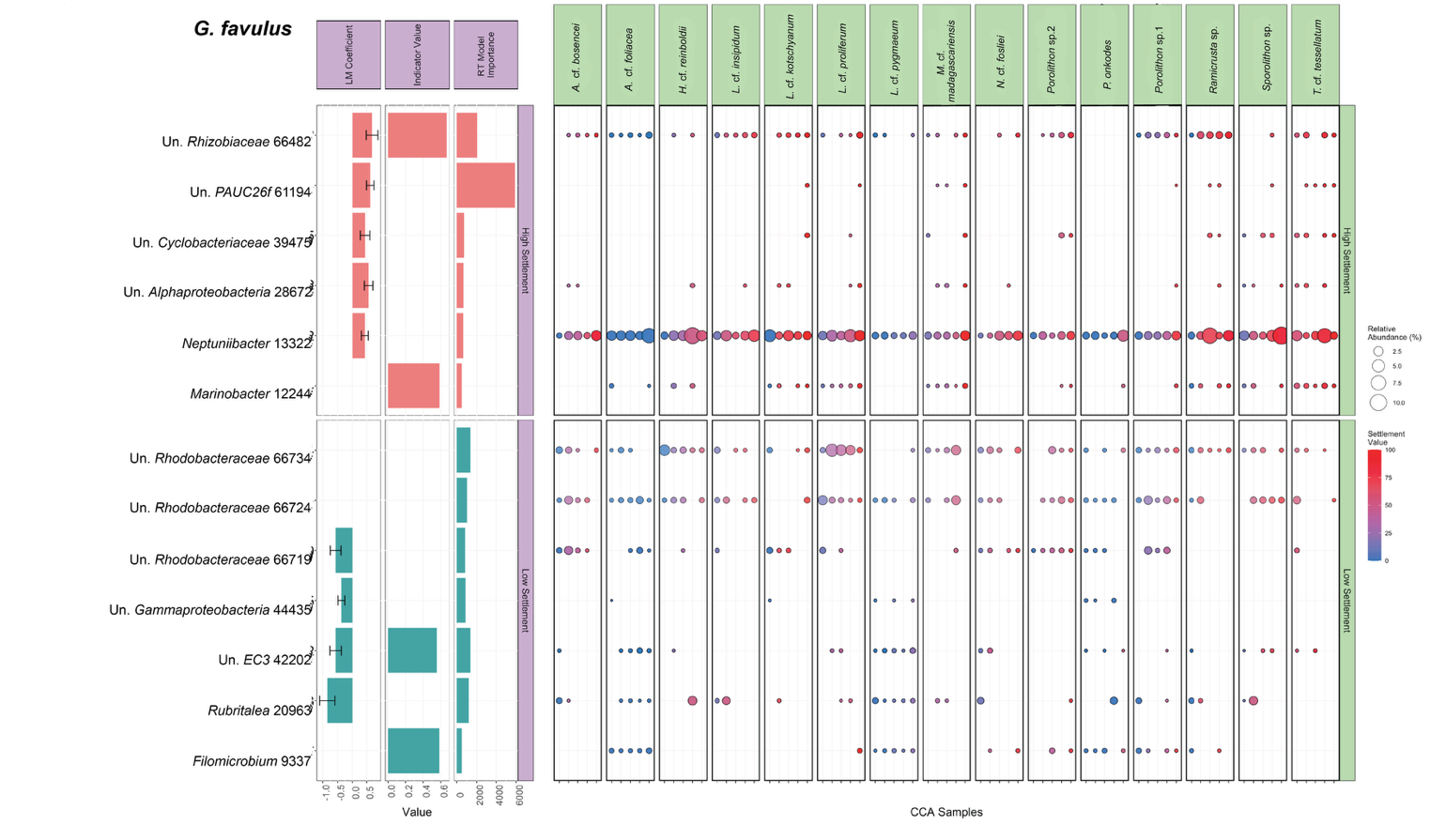
Figure S10. Amplicon sequence variants (ASVs) of interest associated with high and low *F. favulus* settlement. ASVs were deemed of interest if they had significant results in two out of the four methods used (Linear models, differential abundance, indicator species, and random forest analyses). Method results are visualised as bars for each ASV, and the greater the method value the more associated the ASV is with high or low settlement samples. No ASVs were significantly differential abundant in either high or low settlement. ASV distribution is visualised across all samples grouped by crustose coralline algae in the bubble plot. The size of the bubble corresponds to the ASVs relative abundance, and the colour refers to the settlement score in the given sample (Red = high settlement, Blue = low settlement). Each ASV is labeled with an arbitrary number to differentiate individual ASVs across the entire dataset.

***Lobophyllia corymbosa***

There was a significant difference between the high and low settlement crustose coralline algae (CCA) microbial communities (PERMANOVA: F=2.35, p<0.001), with specific amplicon sequence variants (ASVs) found from a combination of linear models, differential abundance, indicator species, and random forest (RF) analyses. There were 15 ASVs associated with high settlement with only an unassigned *Methyloligellaceae* and a *Neptuniibacter* ASV being present in at least one sample from every single CCA species with higher relative abundances in high settlement samples (Figure S11). A majority of the high settlement ASVs were present in high settlement CCA *M.* cf. *madagascariensis*. Furthermore, 17 ASVs were associated with low settlement and were mainly concentrated in low settlement *A.* cf. *foliacea, L.* cf. *pygmaeum, Porolithon* sp.2, and *P. onkodes* samples (Figures S11).


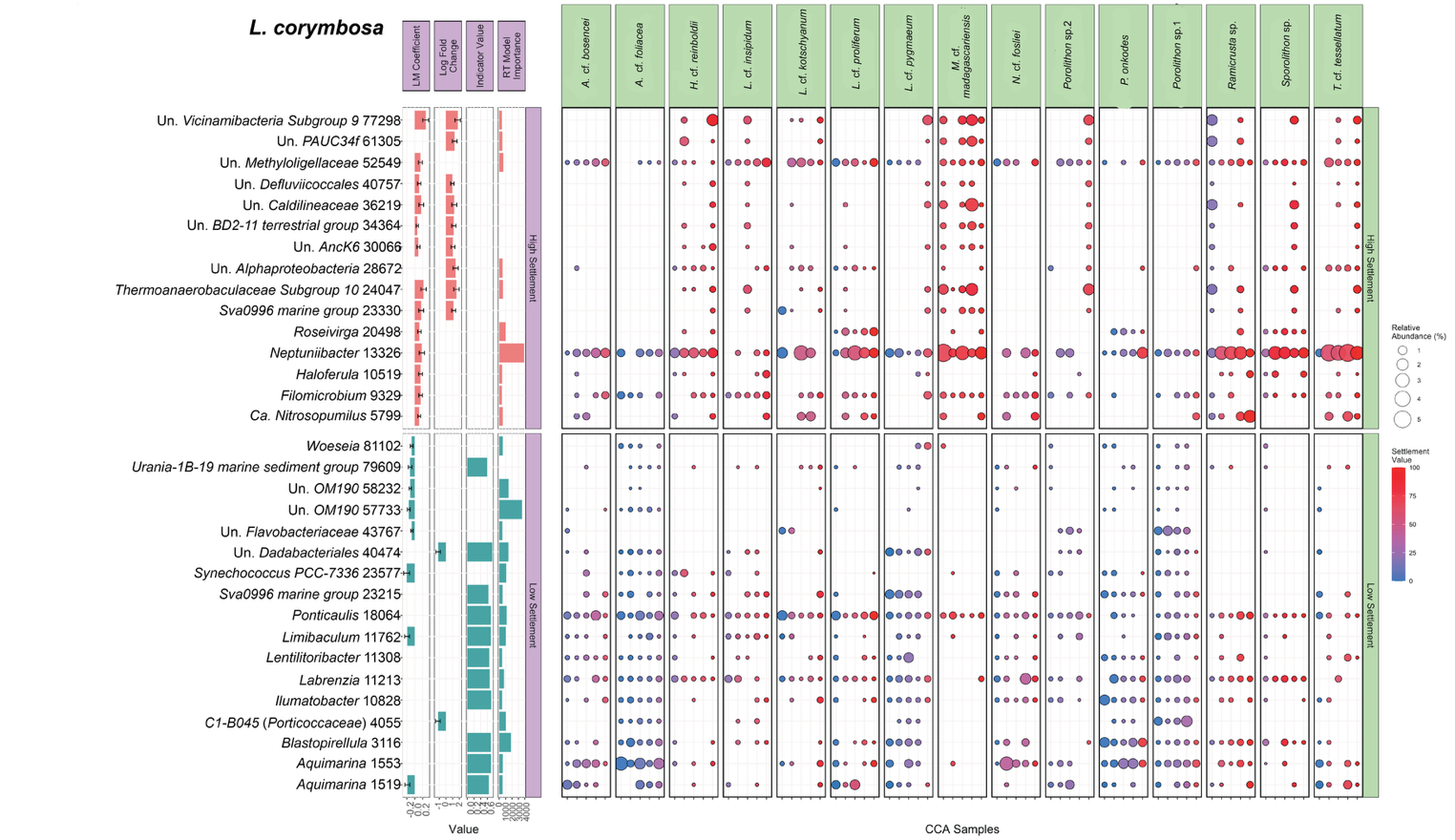


Figure S11. Amplicon sequence variants (ASVs) of interest associated with high and low *L. corymbosa* settlement. ASVs were deemed of interest if they had significant results in two out of the four methods used (Linear models, differential abundance, indicator species, and random forest analyses). Method results are visualised as bars for each ASV, and the greater the method value the more associated the ASV is with high or low settlement samples. ASV distribution is visualised across all samples grouped by crustose coralline algae in the bubble plot. The size of the bubble corresponds to the ASVs relative abundance, and the colour refers to the settlement score in the given sample (Red = high settlement, Blue = low settlement). Each ASV is labeled with an arbitrary number to differentiate individual ASVs across the entire dataset.

***Montipora aequituberculata***

There was a significant difference between the high and low settlement crustose coralline algae (CCA) microbial communities (PERMANOVA: F=2.23, p<0.001), with specific amplicon sequence variants (ASVs) found from a combination of linear models, differential abundance, indicator species, and random forest (RF) analyses. Only one unassigned *Rhodobacteraceae* ASV was found to be associated with high larval settlement, and was only present in *A.* cf. *bosencei, L.* cf. *insipidium, L.* cf. *kotschyanum, Ramicrusta* sp., *Porolithon* sp.1, *Porolithon* sp.2, and *P. onkodes* samples (Figure S12). Eight ASVs were associated with low settlement and consisted of *Rhodobacteraceae, Flavobacteriaceae, Saprospiraceae,* and *Kiloniellaceae* (Figure S12). These ASVs were mainly present in low settlement *A.* cf. *foliacea*, *L.* cf. *proliferum*, and *L.* cf. *pygmaeum* samples, but were sporadically present in low settlement samples of other CCA species.


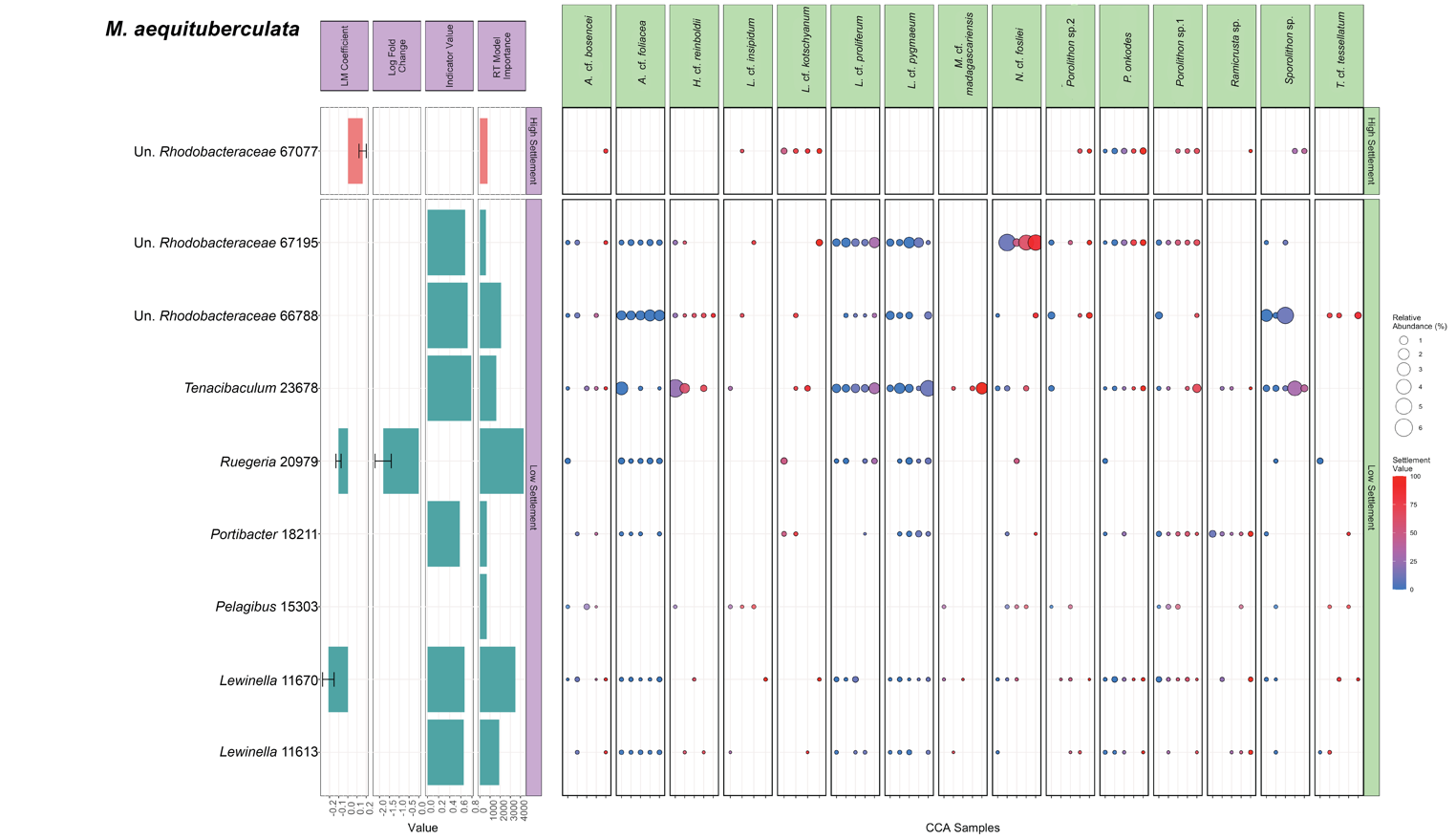


Figure S12. Amplicon sequence variants (ASVs) of interest associated with high and low *M. aequituberculata* settlement. ASVs were deemed of interest if they had significant results in two out of the four methods used (Linear models, differential abundance, indicator species, and random forest analyses). Method results are visualised as bars for each ASV, and the greater the method value the more associated the ASV is with high or low settlement samples. ASV distribution is visualised across all samples grouped by crustose coralline algae in the bubble plot. The size of the bubble corresponds to the ASVs relative abundance, and the colour refers to the settlement score in the given sample (Red = high settlement, Blue = low settlement). Each ASV is labeled with an arbitrary number to differentiate individual ASVs across the entire dataset.

***Mycedium elephantotus***

There was a significant difference between the high and low settlement crustose coralline algae (CCA) microbial communities (PERMANOVA: F=2.56, p<0.001), with specific amplicon sequence variants (ASVs) found from a combination of linear models, differential abundance, indicator species, and random forest (RF) analyses. Eight ASVs including two Neptuniibacter ASVs, were found associated with high settlement (Figure S13). These taxa were largely found in high settlement CCA samples of *H.* cf. *reinboldii, L.* cf. *kotschyanum, L.* cf. *proliferum, M.* cf. *madagascariensis, Ramicrusta* sp., *Sporolithon* sp. and *T.* cf. *tessellatum* (Figure S13). Forty ASVs were associated with low settlement including four *Blastopirellula* ASVs, and all of these ASVs were present in low settlement samples of *A.* cf. *foliacea, L.* cf. *pygmaeum,* and *Porolithon* sp.1 (Figures S13).


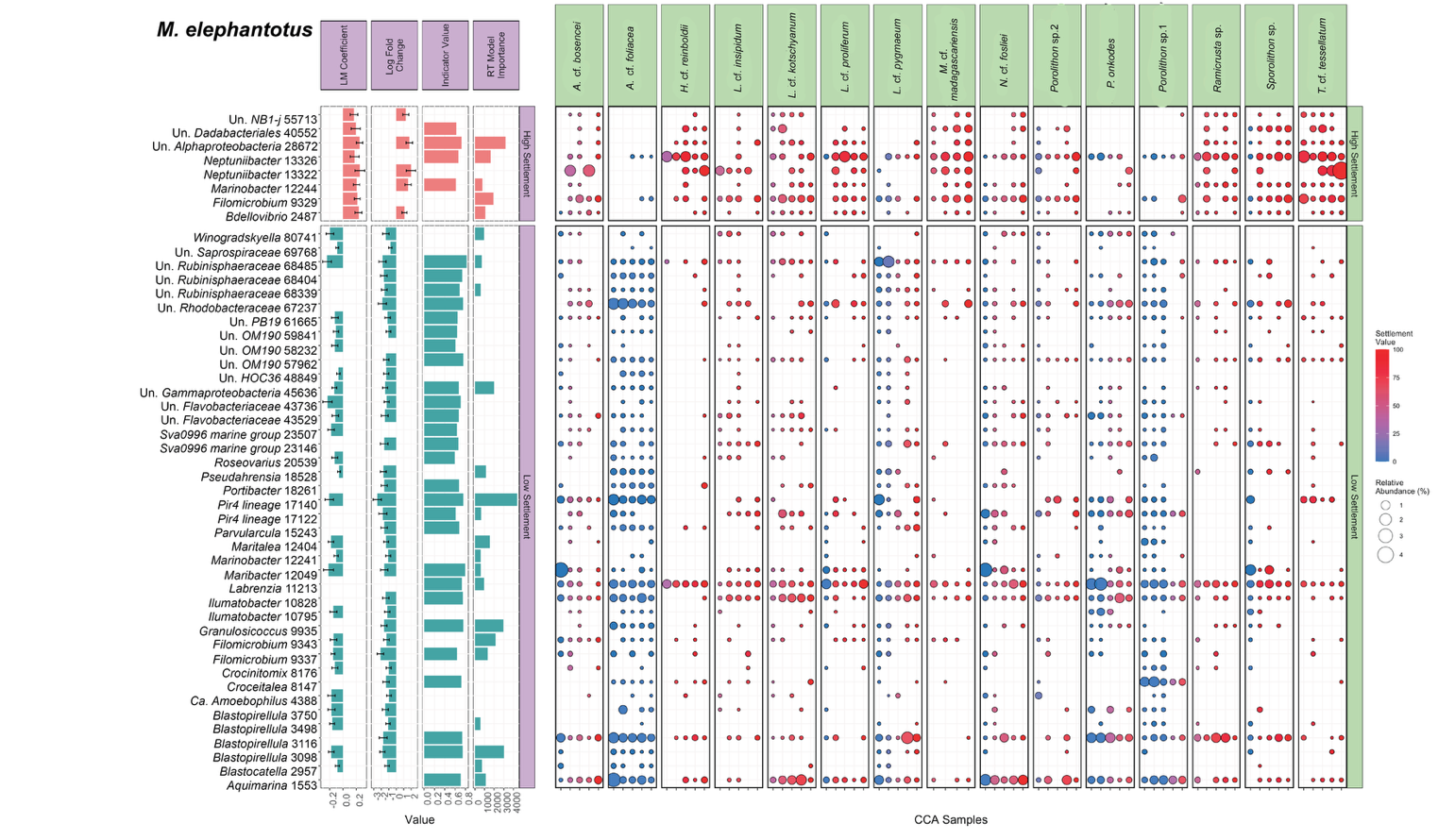


Figure S13. Amplicon sequence variants (ASVs) of interest associated with high and low *M. elephantotus* settlement. ASVs were deemed of interest if they had significant results in two out of the four methods used (Linear models, differential abundance, indicator species, and random forest analyses). Method results are visualised as bars for each ASV, and the greater the method value the more associated the ASV is with high or low settlement samples. ASV distribution is visualised across all samples grouped by crustose coralline algae in the bubble plot. The size of the bubble corresponds to the ASVs relative abundance, and the colour refers to the settlement score in the given sample (Red = high settlement, Blue = low settlement). Each ASV is labeled with an arbitrary number to differentiate individual ASVs across the entire dataset.

***Platygyra daedalea***

There was no significant difference between the high and low settlement crustose coralline algae (CCA) microbial communities (PERMANOVA: F=1.09, p=0.239) and likely not responding to microbial cues. Thirteen ASVs associated with high settlement were identified with having significant results in at least two analyses of linear models, indicator species, differential abundance, and/or random forest analysis, however, the sample distribution between high settlement (n = 68) and low settlement samples (n = 4) is disproportionate and therefore might introduce some bias to these results (Figure S14).


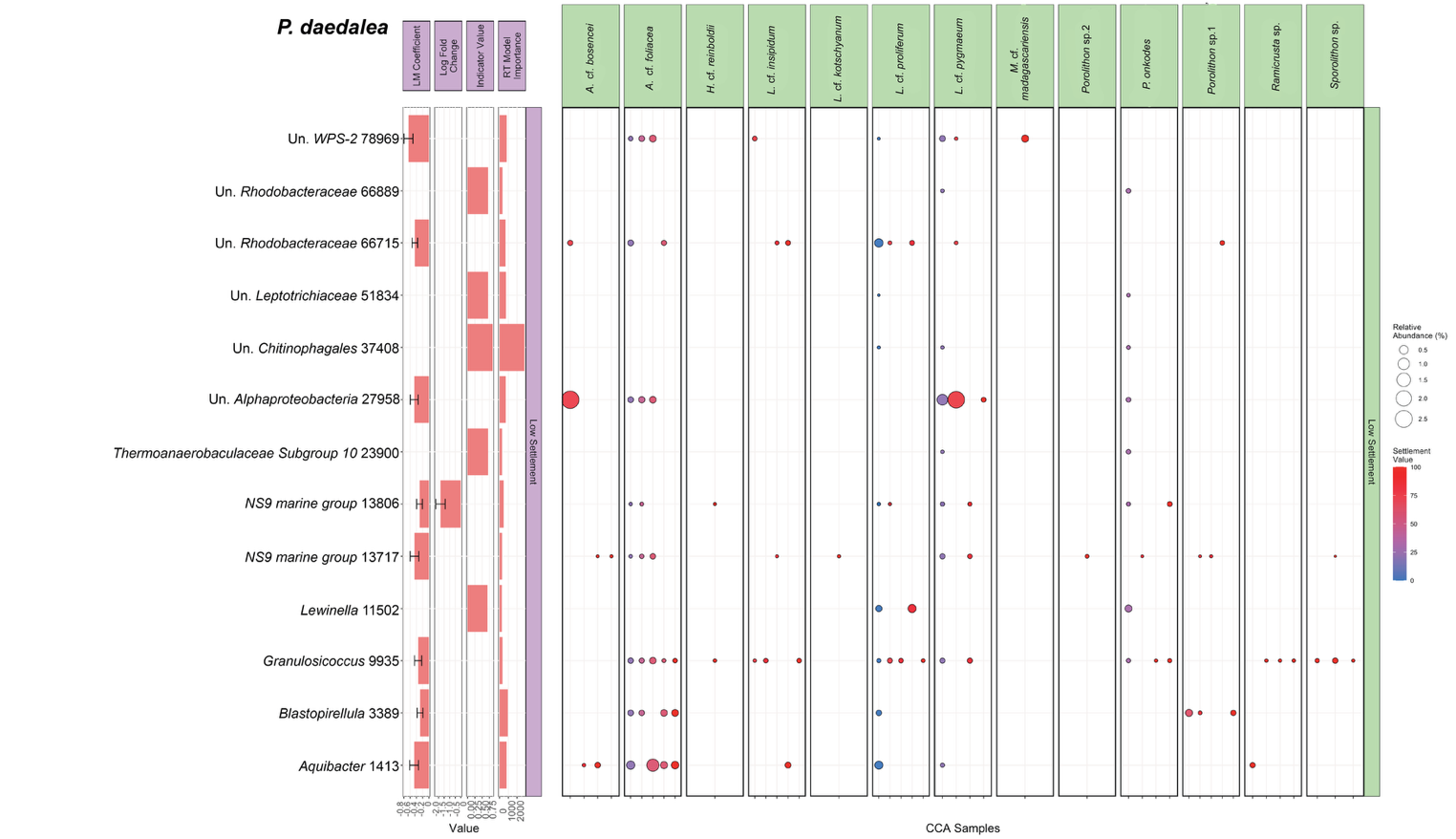


Figure S14. Amplicon sequence variants (ASVs) of interest associated with high *P. daedalea* settlement. ASVs were deemed of interest if they had significant results in two out of the four methods used (Linear models, differential abundance, indicator species, and random forest analyses). Method results are visualised as bars for each ASV, and the greater the method value the more associated the ASV is with high or low settlement samples. ASV distribution is visualised across all samples grouped by crustose coralline algae in the bubble plot. The size of the bubble corresponds to the ASVs relative abundance, and the colour refers to the settlement score in the given sample (Red = high settlement, Blue = low settlement). Each ASV is labeled with an arbitrary number to differentiate individual ASVs across the entire dataset.

***Platygrya sinensis***

There was a significant difference between the high and low settlement crustose coralline algae (CCA) microbial communities (PERMANOVA: F=2.27, p<0.001), with specific amplicon sequence variants (ASVs) found from a combination of linear models, differential abundance, indicator species, and random forest (RF) analyses. Ten ASVs were associated with high settlement, which included ASVs from the taxa *Kiloniellaceae, Nitrincolaceae, Saprospiraceae, Hyphomicrobiaceae, Nitrosopumilaceae, and* an unassigned *Thalassobaculales* (Figure S15). A majority of these ASVs were absent form *A.* cf. *foliacea* and *L.* cf. *pygmaeum* samples, except for a *Neptuniibacter* ASV, which had higher abundances in high settlement samples (Figure S15). Forty-seven ASVs were associated with low settlement and were mainly found in *A.* cf. *foliacea* and *L.* cf. *pygmaeum* CCA samples (Figure S15).


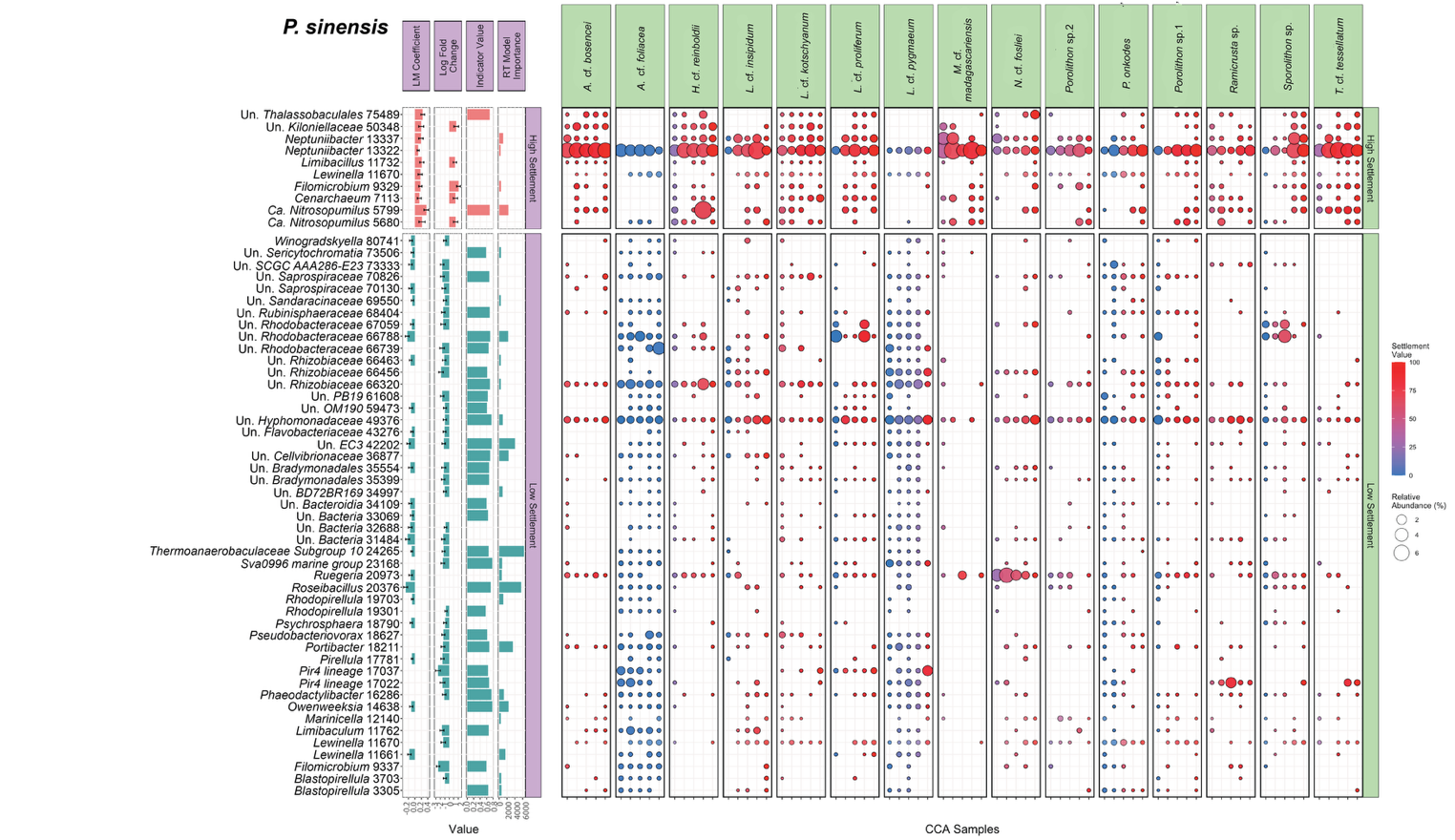


Figure S15. Amplicon sequence variants (ASVs) of interest associated with high and low *P. sinensis* settlement. ASVs were deemed of interest if they had significant results in two out of the four methods used (Linear models, differential abundance, indicator species, and random forest analyses). Method results are visualised as bars for each ASV, and the greater the method value the more associated the ASV is with high or low settlement samples. ASV distribution is visualised across all samples grouped by crustose coralline algae in the bubble plot. The size of the bubble corresponds to the ASVs relative abundance, and the colour refers to the settlement score in the given sample (Red = high settlement, Blue = low settlement). Each ASV is labeled with an arbitrary number to differentiate individual ASVs across the entire dataset.

***Porites lobata***

There was no significant difference between the high and low settlement crustose coralline algae (CCA) microbial communities (PERMANOVA: F=1.14, p=181) and likely not responding to microbial cues. Three ASVs associated with high settlement were only found significant with indicator species and random forest analysis (Figure S16).


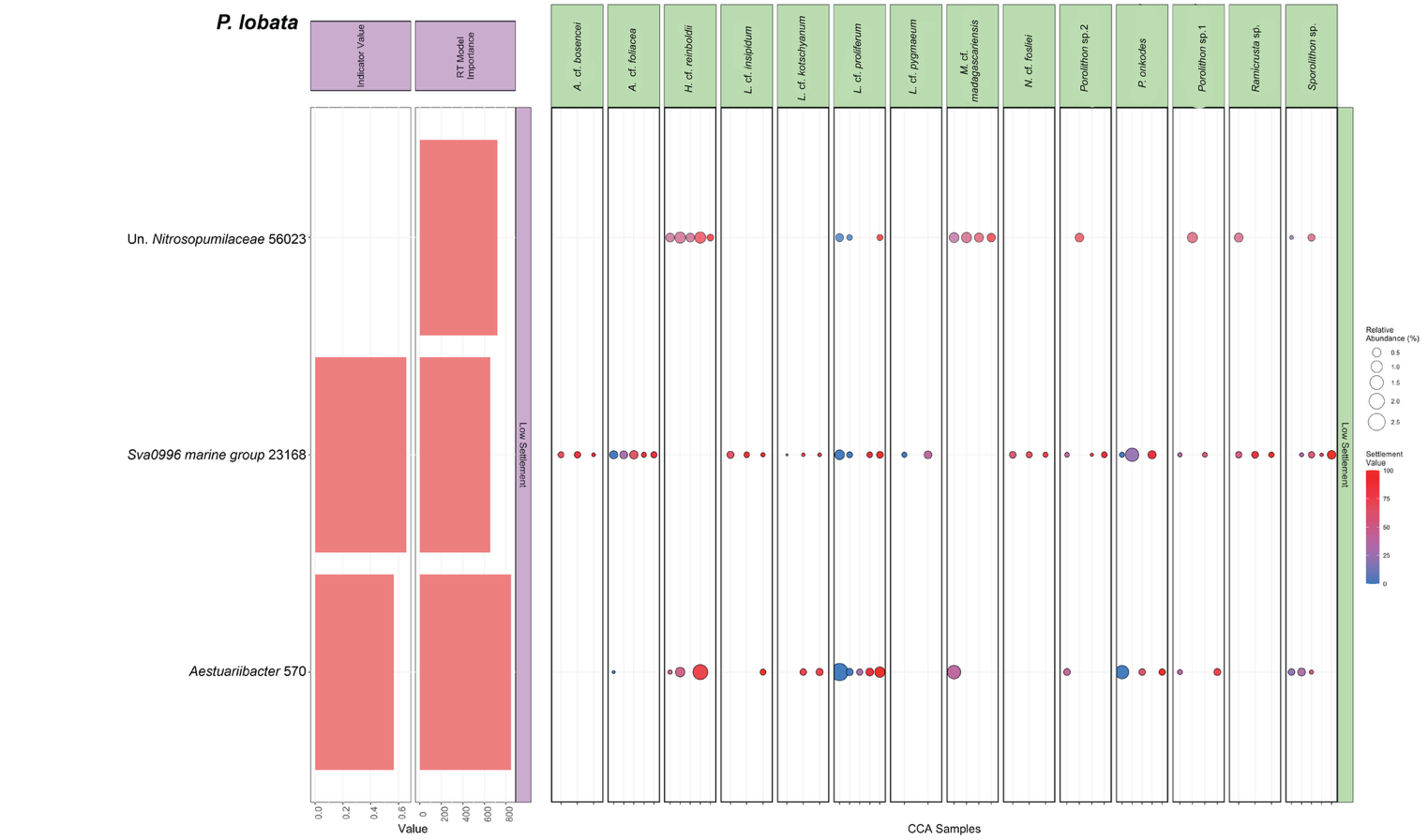


Figure S16. Amplicon sequence variants (ASVs) of interest associated with high *P. lobata* settlement. ASVs were deemed of interest if they had significant results in two out of the four methods used (Linear models, differential abundance, indicator species, and random forest analyses). Method results are visualised as bars for each ASV, and the greater the method value the more associated the ASV is with high or low settlement samples. ASV distribution is visualised across all samples grouped by crustose coralline algae in the bubble plot. The size of the bubble corresponds to the ASVs relative abundance, and the colour refers to the settlement score in the given sample (Red = high settlement, Blue = low settlement). Each ASV is labeled with an arbitrary number to differentiate individual ASVs across the entire dataset. No ASVs were significantly associated with settlement in the linear models and differential abundance analysis.

**References**

1. Abdul Wahab M, Ferguson S, Snekkevik VK, McCutchan G, Jeong S, Severati A, et al. Hierarchical settlement behaviours of coral larvae to common coralline algae. Scientific Reports. 2023;13(1):5795.
